# Luteinizing Hormone-Releasing Hormone (LHRH)-Targeted Treatment in Ovarian Cancer

**DOI:** 10.3390/ijms262411884

**Published:** 2025-12-09

**Authors:** Pallavi Nayak, Michela Varani, Anna Giorgio, Giuseppe Campagna, Donatella Caserta, Alberto Signore

**Affiliations:** 1 Nuclear Medicine Unit, Department of Medical-Surgical Sciences and Translational Medicine, Faculty of Medicine and Psychology, University Hospital Sant’Andrea, Sapienza University of Rome, 00189 Roma, Italy; anna.giorgio@phd.unipd.it (A.G.); giuseppe.campagna@uniroma1.it (G.C.); alberto.signore@uniroma1.it (A.S.); 2 Department of Medical and Surgical Sciences and Translational Medicine, Ph.D. School in Translational Medicine and Oncology, Faculty of Medicine and Psychology, Sapienza University of Rome, 00189 Rome, Italy; 3 Gynecology Division, Department of Medical and Surgical Sciences and Translational Medicine, University Hospital Sant’Andrea, Sapienza University of Rome, 00189 Rome, Italy; donatella.caserta@uniroma1.it

**Keywords:** LHRH analogue, receptor targeting, ovarian cancer, nanotechnology

## Abstract

Ovarian cancer remains one of the most lethal gynecologic malignancies and requires more effective and targeted treatment strategies. Luteinizing hormone-releasing hormone (LHRH), or gonadotropin-releasing hormone (GnRH), receptors are expressed in approximately 80% of ovarian tumors, representing a promising target for targeted drug delivery. This narrative review aimed to explore the development and advancements of LHRH-receptor targeted therapies in ovarian cancer. A bibliographic search was performed using PubMed, Scopus, Google Scholar, and Web of Science. The search strategy included studies on LHRH-peptide drug delivery systems and LHRH-conjugate nanosystems. Literature search covered in vitro studies, preclinical models, and ongoing clinical trials from 2000 to 2025. A total of 19 studies were included for peptide-drug delivery, and 30 studies were included for LHRH-conjugated nanosystems. Overall findings demonstrated enhanced preclinical efficacy, achieving ~50–80% tumor-growth inhibition and 2–4-fold higher cellular uptake, alongside reduced systemic toxicity. Early clinical studies, although limited, reported an overall response/disease-control rate of approximately 50%, supporting improved tumor accumulation of drugs, small interfering RNA (siRNA), and diagnostic agents. Ovarian cancer-specific therapy, targeting LHRH receptors, represents a promising strategy to enhance therapeutic outcomes. Further efforts in preclinical and clinical research are essential to refine personalized treatments and integrate them with a combination of therapies.

## 1. Introduction

Ovarian cancer (OVC) is reported as one of the deadliest gynaecologic malignancies, and its incidence rate is increasing gradually [1,2]. According to the 2022 World Health Organization (WHO) global cancer statistics, 324,603 cases were reported, with a mortality rate of 206,956 for OVC [3]. More than 70% of patients had advanced diseases at presentation due to vague symptoms in an early stage [4]. Although standard treatments for OVC, such as surgery, chemotherapy, a combination of surgery and chemotherapy, and PARP inhibitor targeting, have improved survival, overall efficacy remains limited [5,6,7].

**Figure 1:**
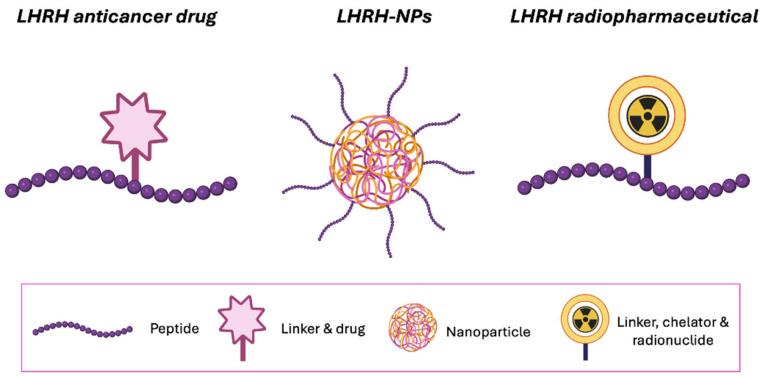
LHRH functionalized approaches to target LHRH-R. (Created in BioRender, https://BioRender.com).

Platinum or paclitaxel (PTX) based regimens are more common in OVC treatment after initial cytoreductive surgery. Even after the improvement of therapy, most patients experience a relapse within three years because of chemoresistance, which leads to a low survival rate [8]. Hormonal treatment also poses challenges, such as the development of resistance and limited efficacy in aggressive subtypes [9]. Overall, side effects and the development of resistance remain major drawbacks. A receptor-targeted treatment approach could be an efficient alternative to overcome these challenges. The receptor targeting strategy has emerged as a powerful approach to enhance the delivery of therapeutic molecules to specific tissues. Over the years, several investigations have been conducted to emphasize this approach for treating solid tumors and circulating cancer cells [10]. The findings suggested that targeting receptors with specific ligands helps to access targeted tumor cells, reduces unwanted off-target effects, and increases the therapeutic efficacy [11,12]. Receptor targeting strategies can be achieved through an active targeting mechanism using specialized drug carriers such as peptides/proteins, nanoparticles (NPs), and radiopharmaceuticals [13,14,15,16,17,18,19] (Figure 1). One of the significant advantages of this approach over non-targeted formulations lies in the systemic administration of targeted formulations, which leads to more accumulation in pathological tissue and less exposure to healthy surroundings [20]. G protein-coupled receptors (GPCRs) are involved in many aspects of tumorigenesis, including the promotion of aberrant growth, increased cell viability, angiogenesis, and metastasis [21]. From the GPCR family, luteinizing hormone-releasing hormone (LHRH), also known as gonadotropin-releasing hormone (GnRH), regulates the production of sex hormones. Beyond its endocrine function, LHRH also utilizes a molecular target, as its receptors (LHRH-R) are overexpressed on the surface of hormone-responsive cancers like breast [22], prostate [23], and ovary. The significance of LHRHR as a tumor suppressor in OVC has been hypothesized because OVC patients with lower tumor expression levels of LHRH demonstrated favorable survival rates. In vitro studies support this, showing that LHRHR agonists like Buserelin and [D-Ala^6^] GnRH inhibit ovarian cancer cell growth in a time- and dose-dependent manner, while the antagonist Antide reverses this inhibition [24].

This narrative review provides a comprehensive overview of the chemistry of LHRH analogues, the pharmacological mechanisms of LHRH receptors, and recent advancements in LHRH peptide engineering for targeted cancer therapy. It further discusses strategies for conjugating LHRH peptides with anticancer drugs and nanocarriers to enhance selectivity and therapeutic efficacy. By integrating findings from both preclinical and clinical studies, this state-of-the-art review aims to inform the design of novel LHRH-based therapeutic molecules and support the development of radiolabelled LHRH conjugates for improved diagnosis and treatment of OVC (Figure 1).

## 2. Search Strategy

A literature search was conducted in databases such as PubMed, Scopus, Google Scholar, and Web of Science, spanning the years 2000 to 2025. The search used a combination of keywords, including Luteinizing Hormone-Releasing Hormone, Gonadotropin-releasing hormone, Anticancer drug, Ovarian cancer, Peptide conjugates, Nanoparticles, and Radiopharmaceuticals. Boolean operators (AND, OR) were applied, and search strings were adapted for each database. The bibliography of the research articles was manually searched to assess further relevant articles. Both preclinical and clinical studies were included. Articles, such as letters to the editor, supplementary commentaries, and non-English publications, were excluded.

## 3. LHRH and Its Analogue

**Figure 2:**
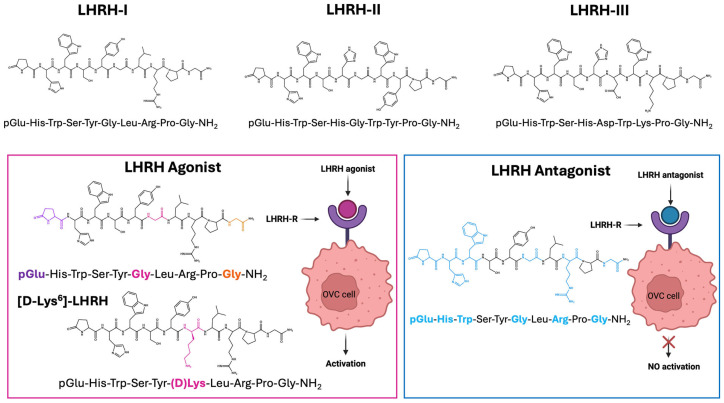
Structure of native LHRH and its analogue.

Three types of LHRH have been reported in vertebrates. LHRH-I (known as mammalian LHRH), LHRH-II (initially discovered in chicken brain), and LHRH-III (isolated from lampreys) [25]. These peptides have a similar structure, consisting of 10 amino acids, but differ in specific residues (Figure 2) [26,27].

LHRH-I, the isoform found in mammals, is a decapeptide (pGlu-His-Trp-Ser-Tyr-Gly-Leu-Arg-Pro-Gly-NH2) responsible for controlling the hypothalamic pituitary gonadal axis and for regulating reproductive activities in animals.

Native LHRH-I is naturally unstable and rapidly degraded in vivo, limiting its direct use for targeting LHRH-R in therapeutic applications. For this reason, synthetic LHRH analogues (LHRHa) have been developed by modifying specific amino acid residues to enhance peptide stability, receptor affinity, and biological activity [28,29,30,31].

These analogues include both agonists and antagonists. LHRH agonists initially stimulate the release of gonadotropins (LH and FSH) but subsequently cause their downregulation. The synthetic analogues are designed with several structural modifications to enhance their pharmacological properties. These include the substitution of glycine at position 6 with a D-amino acid to increase receptor affinity and resistance to enzymatic degradation by peptidases. Additionally, the replacement of the Gly-NH_2_ group at position 10 in the C-terminal region with more hydrophobic moieties, such as ethylamide or azaglycine-amide, extends the plasma half-life of the peptide. Modifications to the pyroglutamic acid (pGlu) at the N-terminal further improve metabolic stability and reduce degradation by aminopeptidases, enhancing the overall efficacy and duration of action of LHRH agonists (Figure 2).

In contrast, LHRH antagonists bind competitively to the LHRH receptor without triggering its activation, resulting in immediate inhibition of gonadotropin release and a more rapid suppression of sex hormone production.

Modifications in LHRH synthetic antagonists involve the substitution of multiple amino acid residues at positions 1, 2, 3, 6, 8, and 10 with hydrophobic groups, such as D-2-naphthylalanine (D-Nal(2)) and D-3-pyridylalanine (D-Pal(3)). These alterations enhance resistance to enzymatic degradation, increase receptor binding affinity, and reduce immunogenicity (Figure 2). These modifications not only extend the plasma half-life of the peptide but also shift its function from a receptor agonist to a potent competitive antagonist [32].

The improved pharmacokinetic properties of these synthetic analogues allow for the selective targeting of LHRH-Rs, which are overexpressed in various hormone-dependent cancers. This high receptor specificity has led to the development of LHRH-based peptide-drug conjugates as promising agents for targeted cancer therapy. These conjugates are currently being investigated for the treatment of a wide range of malignancies, including breast and prostate cancer, melanoma, soft tissue sarcomas, and other solid tumors [29].

Therapeutic peptides, including LHRHa, are typically composed of well-defined sequences of amino acids, with molecular weights ranging from 500 to 5000 Da. Their flexibility, high specificity, and low toxicity make them attractive platforms for the development of targeted therapies.

## 4. LHRH-Receptors and Their Pharmacological Mechanism Overview

**Figure 3:**
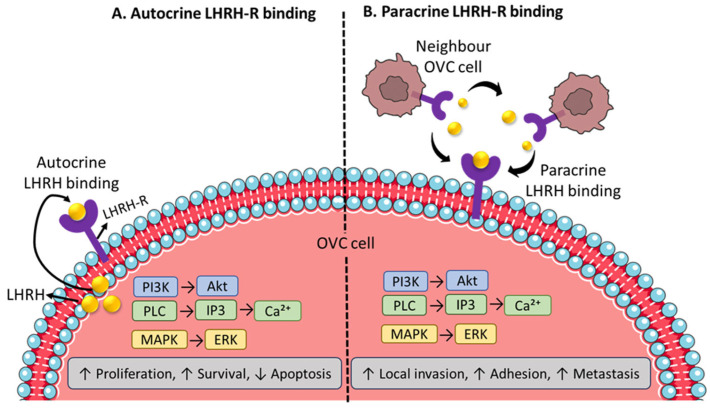
Autocrine vs. paracrine LHRH-R signaling. (Created in Bioicons, https://bioicons.com/).

LHRH-R is significantly overexpressed in hormone-responsive cancers, including breast (~50%), endometrial (~80%) [33,34], prostate (~86%) [35,36], and ovarian cancers (~90%) (Figure 3B) [37]. Although LHRH receptors are common in epithelial OVC, their expression patterns are heterogeneous across tumor types and experimental models. Völker et al. used radioligand binding and RT-PCR to discover high-affinity LHRH binding sites and LHRH-R mRNA in 70% of primary epithelial ovarian malignancies and 83% of endometrial cancers, indicating the presence of active pituitary-type LHRH receptors in the majority of tumors [38]. Immunohistochemical profiling across OVC histotypes has shown distinct patterns of LHRH-R expression between tumor subgroups. In high-grade serous ovarian carcinoma (HGSC), nearly 90% of cases were LHRH-R-positive (LHRH-R^+ve^), while low-grade serous tumors showed receptor expression in essentially all samples (100%), and about 82% of high-grade serous tumors were positive in an independent cohort [39].

In healthy cells, LHRH binds to LHRH-R on gonadotroph cells in the pituitary gland, activates Gq/11 proteins, which stimulate phospholipase C (PLC), leading to the activation of the second messenger pathway IP3/DAG [40,41].

IP3 induces the release of calcium (Ca^2+^) from intracellular deposits, while DAG+, in combination with Ca^2+^, activates PKC, promoting the secretion of LH and FSH (Figure 3A). LHRH agonists stimulate LH and FSH by binding to LHRH-R in the pituitary gland; however, prolonged exposure renders the receptors less sensitive, leading to downregulation. In contrast, LHRH antagonists bind directly to LHRH-R and immediately block natural LHRH from binding, thereby suppressing LH and FSH secretion without causing the initial hormonal release (Figure 3) [42].

In tumor cells, LHRH-R does not regulate LH or FSH release, but is involved in autocrine and paracrine signaling pathways that promote cancer cell survival and progression (Figure 3). In the autocrine pathway, tumor cells produce LHRH, which binds to LHRH-R on the same cell, activating intracellular signaling such as PI3K/Akt, PLC/IP3/Ca^2+^, and MAPK/ERK, and promoting cell growth and preventing cell death [28,43].

In the paracrine mechanism, tumor cells release LHRH into the tumor microenvironment, where it binds to LHRH-R on nearby tumor cells, facilitating cell adhesion and metastasis. LHRH-R is also overexpressed in several non-hormone-responsive cancers, such as the brain [44], kidney [45], liver [46], skin [47], and pancreas [48].

Due to its limited expression in normal tissues and high prevalence in tumors, LHRH-R has been effectively studied for targeted imaging and therapeutic applications by conjugating LHRHa with diagnostic and cytotoxic agents, enabling selective delivery to LHRH-positive tumors [49].

## 5. LHRH Peptide Engineering for Targeted Delivery

**Figure 4:**
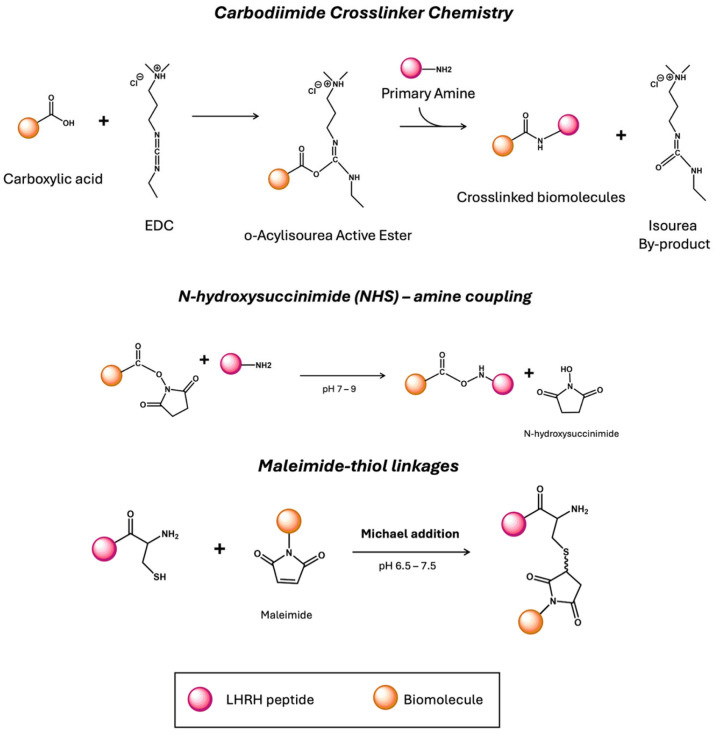
Conjugation methods to attach LHRH peptide to biomolecules.

Several studies have been conducted on LHRH peptide engineering for targeted delivery, focusing on the conjugation of therapeutic and imaging agents to LHRH analogues. The primary approach of this method is to ensure selective delivery of these conjugates to tumor cells overexpressing LHRH-R, minimizing uptake by normal tissues with low or no receptor expression. [D-Lys^6^]-LHRH has been the most commonly used analogue due to its high binding affinity to LHRH-R [10]. Covalent conjugation methods, such as 1-ethyl-3-(3-dimethylaminopropyl)carbodiimide hydrochloride (EDC)/N-hydroxysuccinimide (NHS) chemistry and maleimide-thiol linkages, are widely used to link drugs or imaging agents to the peptide (Figure 4) [50,51]. In addition, peptides can be radiolabelled with several radioisotopes for diagnostic (^99m^Tc, ^68^Ga) or therapeutic (^177^Lu) purposes, though different bifunctional chelators like DOTA, NOTA, or HYNIC [52,53,54].

Despite their promise, LHRH peptide conjugates face several challenges, including limited stability, potential immunogenicity, a short circulation time, and rapid enzymatic degradation, which limit their in vivo distribution and therapeutic window. To address these limitations, conjugation with nanosystems has emerged as a promising strategy to improve pharmacokinetic profiles, enhance stability, and significantly prolong the half-life of the peptide conjugates [55].

## 6. LHRH-Conjugated Anticancer Drugs for Targeting Therapy

**Figure 5:**
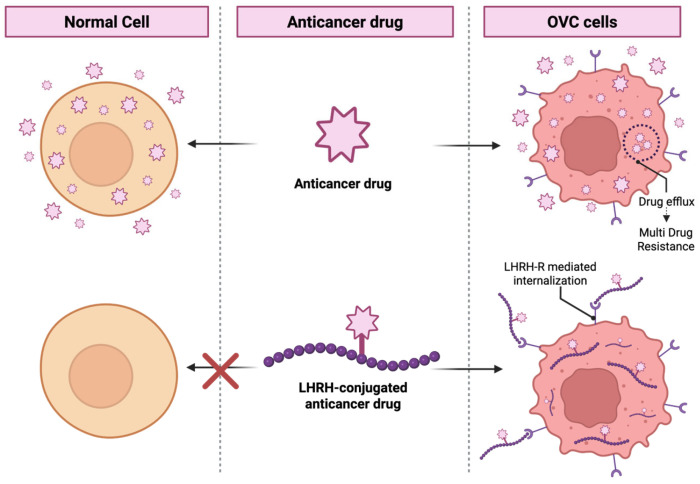
Response of normal and OVC cells to anticancer drug vs. LHRH-conjugated anticancer drug.

Several LHRHa-anticancer drug conjugates have been developed and evaluated for their potential to target OVC cells. Among these, cisplatin, melphalan, and anthracycline have been conjugated to [D-Lys^6^]-LHRH, resulting in effective cancer cell targeting and cytotoxicity (Figure 5) [56].

### 6.1. Preclinical Studies

In preclinical studies, multiple LHRHa conjugated formulations were evaluated on OVC cell lines. The results showed receptor-dependent uptake and antitumor activity in LHRH-R^+ve^ cell lines, and a diminished effect in LHRH-R-negative (LHRH-R^−ve^) cell lines (Table 1).

#### 6.1.1. Anthracycline Conjugates

The anthracycline, such as doxorubicin (DOX), is a commonly used chemotherapeutic agent. However, the administration of free anticancer drugs poses challenges like multidrug resistance, toxicity to healthy cells, and a lack of tumor selectivity. Peptide-drug conjugates can overcome these challenges by selectively binding carrier molecules to specific binding sites on cancer cells. A DOX molecule was conjugated to the LHRH analogue [D-Lys^6^]-LHRH to produce the cytotoxic compound AN-152/AEZS-108, which exhibits high affinity for LHRH receptors and strong anticancer activity. Studies have collectively evaluated the antiproliferative effects, apoptotic effects, and receptor-mediated internalization of AN-152 and DOX in LHRH-R^+ve^ (EFO-27, EFO-21, OVCAR-3) and LHRH-R^−ve^ (SK-OV3) cell lines. The potency (EC_50_ ratio) of DOX and AN-152 was equal in both LHRH-R^+ve^ cells, while in LHRH-R^−ve^ cells, AN-152 showed less potency than DOX. Confocal laser-scanning microscope detection confirmed the presence of AN-152 in the nucleus of LHRH-R^+ve^ and its absence in LHRH-R^−ve^ cells, which implied that AN-152 had a selective receptor-mediated activity in receptor-positive cell lines [57,58]. In LHRH-R^+ve^ cells, AN-152 significantly increased apoptosis compared to DOX alone, but DOX was more effective in LHRH-R^−ve^ cells, indicating the receptor-specific activity of AN-152 [59]. In vivo analysis using the OVC xenograft mouse model (OVCAR-3, SK-OV-3, and ES-2) demonstrated the receptor-dependent efficacy of AN-152. In LHRH-R^+ve^ tumor, AN-152 significantly reduced tumor volume at both low and high doses. While free DOX inhibited tumor growth but did not reduce its volume, even higher doses led to a high mortality rate, while lower doses only caused weight loss. AN-152 did not affect the growth of SK-OV-3 tumors [60,61].

Although primarily focused on receptor-targeted strategies, one study showed that glycolysis inhibition with 2-deoxy-D-glucose further enhances the cytotoxicity of AN-152, suggesting that metabolic co-targeting can amplify the therapeutic effect of receptor-mediated delivery [62].

Following the promising results obtained with AN-152, researchers also synthesized ultra-potent conjugate AN-207. In which the 2-pyrrolino-DOX (AN-201) is conjugated to the ε-amino group of lysine residue on [D-Lys^6^]-LHRH [57]. This modification aimed to improve antitumor efficacy. Arencibia et al. compared AN-207 with its free drug (AN-201) in LHRH-R^+ve^ ES-2 cells and LHRH-R^−ve^ UCI-107 cells. In Es-2 cells, AN-207 at 2–10 nM induced dose-dependent cell death, whereas at ≤1 nM had no effect. UCI-107 cells showed minimal response at ≤10 nM, confirming poor uptake in LHRH-R^−ve^ cells. However, higher doses caused some non-specific toxicity due to the slow release of AN-201. These findings highlight the receptor-mediated targeting of AN-207 with limited off-target effects at lower concentrations [63]. Following significant results from in vitro investigations, the Arencibia team conducted an additional study to investigate the impact of AN-207 and AN-201 on the ES-2 tumor model. Three in vivo investigations were performed. In the first experiment, AN-207 reduced tumor growth by 59.5%, whereas AN-201 and [D-Lys^6^]-LHRH had no significant effect, indicating the need for chemical conjugation for receptor-mediated targeting. In the second experiment, AN-207 also suppressed the growth of larger tumors more effectively than AN-201. The third experiment demonstrated that inhibiting LHRH-R with Cetrorelix significantly reduced the anticancer efficacy of AN-207 while increasing systemic toxicity, indicating LHRH-R-specific uptake [64]. Buchholz and coworkers conducted the animal study using the OV-1063 and ES-2 tumor mouse models. Here, the treatment group received a combination of AN-207 and somatostatin analogues (AN-238). The results demonstrated a 50–60% reduction in tumor volume in both models; however, AN-201 did not effectively inhibit tumor growth [65].

#### 6.1.2. Lytic Peptide Conjugates

Esperance Pharmaceuticals developed EP-100, a combination of a cationic α-helical lytic peptide (CLIP-71) with a natural LHRH ligand. EP-100 is a fusion peptide designed to deliver lytic peptides to tumor cells via LHRH-R. EP-100 exhibited a high anticancer effect in LHRH-R-expressing tumors, both alone and in combination with anticancer drugs [66]. Ma et al. investigated the synergistic effect of EP-100 with five different anticancer drugs (PTX, DOX, cisplatin, olaparib, and topotecan). They showed a high synergistic effect with olaparib. The researchers then conducted in vitro tests using EP-100 alone and in combination with olaparib in eight OVC cell lines, as well as animal studies in HeyA8 (BRCA wild-type) models. The results showed that the IC_50_ of EP-100 alone ranged from 0.80 to 2.56 µM, indicating significant cytotoxicity. However, LHRH-R knockdown boosted the IC_50_ value, confirming the receptor-dependent activity. EP-100 alone caused necrotic cell death by rupturing cell membranes via its lytic peptide; however, when combined with olaparib, it exhibited synergistic cytotoxicity in BRCA1/2 wild-type cell lines, such as HeyA8, A2780ip2, OVCAR4, and OVCAR8. A combination of EP-100 and olaparib significantly decreased the tumor weight in the xenograft tumor model by approximately 50% [66].

#### 6.1.3. Other Anticancer Drug Conjugates

Other anticancer drugs, such as camptothecin, mitoxantrone, cisplatin, etc., have been conjugated to LHRH peptides or multivalent polymer–peptide constructs to enhance selectivity for OVC cells. Dharap et al. evaluated the dual targeting (LHRh-BH3) with camptothecin (CPT) through a poly(ethylene glycol) (PEG) carrier in A2780 OVC cells. CPT-PEG-LHRH and CPT-PEG-BH3 lowered IC_50_ to picomolar levels in A2780 OVCcell lines, demonstrating a substantial improvement in efficacy. The combination of CPTPEG-BH3 and CPT-PEG-LHRH conjugates had cytotoxicity comparable to CPT-PEGBH3 or CPT-PEG-LHRH conjugates alone [67]. Another group, Chandna et al., doubled the active component (2XCPT-PEG-BH3) and analyzed it in OVC patient-derived primary tumor, malignant ascitic cells, and xenograft tumor model. The findings suggested that doubling the active components increased efficacy and reduced off-target toxicity [68]. Khandare et al. covalently conjugated 1–3 molecules of LHRH peptide and CPT with PEG polymer to evaluate in vitro and in vivo outcomes in A2780 OVC cells and athymic mice model. Cellular uptake analysis revealed that 3 LHRH peptide-PEG-CPT exhibited higher uptake compared to native PEG-CPT and CPT alone. LHRH-PEG-CPT showed 77 times more cytotoxicity than native PEG-CPT. 3×CPT-PEG-3×LHRH conjugate exhibited the most potent tumor suppression [69]. Yao et al. studied the cytotoxicity effect of LHRH-conjugated platinum(IV) prodrug derived from cisplatin (_C_DDP). The peptide-conjugated sample showed significantly higher selectivity towards LHRH-R^+ve^ cells compared to cisplatin alone. In LHRH-R^+ve^ cells, IC_50_ values were 5–8 times higher than in LHRH-R^−ve^ cells. The peptide conjugation demonstrated significantly higher water solubility and tumor selectivity than cisplatin [70]. Markatos et al. fabricated two compounds, Con-3 and Con-7, by conjugating mitoxantrone with modified LHRH peptide analogues (Con-P2 and Con-P1, respectively). These conjugates were expected to selectively transport mitoxantrone to OVC SKOV-3, where the intracellular thioredoxin system would bind and release the active drugs. Both conjugates exhibited substantial antiproliferative effects on SKOV-3 OVC cells, suppressing cell proliferation in a dose- and time-dependent manner, with IC_50_ values ranging from 0.6 to 0.9 µM. Furthermore, they triggered strong apoptosis, with ~50% apoptotic cell death by day 4, similar to the impact of free mitoxantrone [71].

**Table 1:** Overview of preclinical studies on LHRH-conjugated chemotherapeutics for OVC targeting therapy.

Conjugates /Peptide: Drug	Preclinical Details					Ref.
	In Vitro Cell Lines (EC_50_ Ratio)	In Vivo Model (Cell Type)	In Vivo Outcomes (Median Survival or Tumor Size Reduction)	Treatment Group/Dosage/Route of Injection	Key Findings	
AN-152 (D-Lys^6^-LHRH+ DOX)	EFO-21(1.13 ± 0.06), EFO-27 (1.16 ± 0.16), SKOV-3 (1.57 ± 0.06)	NA	NA	AN-152 or DOX (0.3–100 nM depending on cell line); ±10 μM [D-Trp^6^]-LHRH preincubation	LHRH-R^+ve^ (EFO-21, EFO-27): Receptor-mediated actions and intranuclear uptake of the AN-152 LHRH-R^−ve^ cells (SKOV-3): AN-152 less active than DOX	[58]
AN-152 (D-Lys^6^-LHRH+ DOX)/1:1	NA	NIH:OVCAR-3 or SK-OV-3 tumor model	OVCAR-3: AN-152 tumor size reduced to 63–67% of baseline vs. 231–238% in controls. SK-OV-3: No reduction (267% vs. control 275%).	AN-152: 300 or 700 nmol/20 g IV (single dose); DOX equimolar; saline control	The tumor volumes of NIH: OVCAR-3 cancers were reduced significantly 1 week after treatment with AN-152 at both high and low doses	[59]
AN-152 (D-Lys^6^-LHRH+ DOX)/1:1	EFO-21, NIH: OVCAR-3, SKOV-3 cells EC_50_: NA	NA	NA	AN-152 or DOX (1–100 nM, 72 h), ± chloroquine (30 µM) or DFP (0.5 mg/mL)	The apoptotic effect of AN-152 is more selective and effective in LHRH-R^+ve^ cells.	[60]
AN-152 (D-Lys^6^-LHRH+ DOX)/1:1	NA	ES-2 tumor model	34.5% tumor size reduction with AN-152; DOX 16.3% (NS)	AN-152, DOX, and control/345 nmol/20 g BW/IV	Higher percentages of tumor reduction were found in the AN-152 treatment group than in the DOX.	[61]
AN-152 (D-Lys^6^-LHRH+ DOX)/1:1	EFO-21, OVCAR-3 cells EC_50_:NA, % cell viability shown in dose–response curves	NA	NA	2DG 5–20 mM; AN-152 10^−9^–10^−5^ M; GnRH-II antagonist 10^−9^–10^−7^ M; 96 h exposure; single and combination treatments	Combining a 2DG with LHRH-R-targeted therapy significantly improves apoptosis and decreases viability in LHRH-R^+ve^ OVC cells.	[62]
AN-207 ([D-Lys^6^]-LHRH + 2-pyrrolino-DOX (AN-201)	ES-2, UCI-107 cells EC_50_:NA, time- and dose-dependent cytotoxicity shown	NA	NA	ES-2 and UCI-107 cells to AN-207 or AN-201 at 0.1–100 nM (Exp I) or 2–8 nM (Exp II) for 30–240 min.	AN-207 caused cell death in a concentration- and time-dependent manner in ES-2 cells, but not in UCI-107 cells	[63]
AN-207 ([D-Lys^6^]-LHRH + AN-201)/1:1 Blocker: cetrorelix	NA	ES-2 tumor model	AN-207 produced 50–60% tumor inhibition; AN-201 minimally effective; Cetrorelix blocked AN-207 activity	AN-207 (250 nmol/kg IV single or two doses); AN-201 (250 nmol/kg); Cetrorelix 200 µg/mouse SC (blockade)	An unconjugated mixture of drug and peptide was not as effective, whereas AN-207 showed significant inhibition even in a large tumor (400 mm^3^).	[64]
AN-207, AN-215, AN-238 (LHRH/bombesin/somatostatin) linked to AN-201	NA	UCI-107, OV-1063, ES-2 tumor model	Significant tumor inhibition: 36–75% depending on analogue and model; combinations being the strongest; AN-201 ineffective and toxic	IV doses: 150–200 nmol/kg (single or multiple cycles); combinations at 50% dose each	A combination of LHRH with somatostatin reduced tumor growth by 50–60% in both tumor models, while alone, AnN-201 was not so effective.	[65]
EP-100 (GnRH ligand fused to CLIP-71 lytic peptide)	Ovarian cancer cell lines (n = 12); BRCA1/2 mutant and Wild type/EP-100 IC_50_ = 0.80–2.56 µM	OVCAR5 IP model; HeyA8 IP and SC models	EP-100 reduces tumor weight; EP-100 + Olaparib produces near-complete suppression (0.06 g)	EP-100: 0.02–1 mg/kg IV twice weekly; Olaparib: 50 mg/kg IP daily	The synergistic effect of EP-100 with olaparib and EP-100 sensitizes BRCA wild-type OVC cells to olaparib.	[66]
CPT–PEG, CPT–PEG–BH3, CPT–PEG–LHRH/1:1	A2780 cells IC_50_ shift: nanomolar to picomolar	NA	NA	CPT-equivalent 3 nM; 48 h exposure; comparison across free CPT and three conjugates	A successful dual-targeting approach that uses BH3 to overcome apoptosis resistance and LHRH for receptor-mediated delivery.	[67]
CPT–PEG–LHRH–BH3 and 2×CPT–PEG–2×LHRH–2×BH3	Primary OVC tumor and malignant ascitic cells	Female athymic mice; SC tumors from primary or ascites-derived cells	Primary tumors: strong regression with 2×CPT–PEG–2×LHRH–2×BH3; Ascites tumors: growth stabilized	CPT-equivalent 10 mg/kg, IP, 6 doses over 3 weeks	Doubling the active components increased the efficacy and reduced off-target toxicity.	[68]
1–3 CPT + 0–3 LHRH copies on PEG	A2780 cells	A2780 tumor model, tumor ~1 cm^3^ at treatment	3CPT-PEG-3LHRH produced the strongest tumor reduction vs. all other versions	CPT-equivalent 10 mg/kg single IP injection	3×CPT-PEG-3×LHRH exhibited higher cellular uptake, the most potent cytotoxicity, and tumor suppression	[69]
LHRH-Pt (IV)	A2780 ~9–12 µM; SKOV3 76 µM; SI = 8.2; Highly selective	NA	NA	0–100 µM (IC_50_); 10 µM (uptake/DNA); IC_70_ used for apoptosis/cell cycle	LHRH-Pt (IV) prodrugs to selectively target and kill LHRH-R-overexpressing cancer cells, while posing less toxicity to normal cells.	[70]
Con-3/Con-7 LHRH analogues linked to mitoxantrone via disulfide bonds	SKOV-3 Cells EC_50_:Con-3 = 0.78 nM; Con-7 = 1.8 nM	NA	NA	IV, 0.1 nM–10 µM (proliferation), 1 µM (apoptosis/uptake), cisplatin 30 µg/mL	Con-3 and Con-7 Significantly Inhibit SKOV-3 Cell Proliferation and Induce Apoptosis in a Time-Dependent Manner	[71]

### 6.2. Clinical Studies

LHRH-R targeted therapies have been investigated clinically in patients with recurrent or platinum-resistant OVC, aiming to improve tumor-specific delivery (Table 2). Verschraegen et al. studied the anticancer activity of LHRH-R agonist cetrorelix in 17 platinum-resistant OVC patients. The study reported a partial response (18% rate) in 3 patients and a 35% stable disease (SD) rate in 6 patients, indicating a 53% clinical benefit rate (CBR). A biopsy sample from six out of seven patients showed LHRH-R expression, with a correlation between receptor expression and response to therapy, indicating a receptor-mediated action. The treatment reported low toxicity, while only one patient experienced a grade 4 histamine reaction. Overall, this study concluded that cetrorelix had moderate antitumor efficacy and less toxicity [72].

Emons et al. explored the therapeutic potential of LHRH agonist AN-152 in 42 platinum-resistant OVC patients. Patient outcome showed a 52.4% clinical benefit rate, with no complete responses observed. Treatment was well tolerated, with hematologic toxicities such as leukopenia and febrile neutropenia occurring in a small subset of patients. No significant cardiac events were reported [73].

Curtis et al. conducted a Phase I trial to evaluate EP-100 in patients with LHRH-R-expressing solid tumors. Among the seventeen OVC patients, 65% (n = 11) showed LHRH-R positivity, and eight patients were treated with EP-100. In contrast, no objective tumor shrinkage was observed. Two patients (at doses of 2.6 and 11.7 mg/m^2^) achieved disease stabilization for at least 16 weeks. These findings suggest EP-100 may provide a potential clinical benefit in LHRH-R+ve OVC patients with a history of multiple prior treatments [74].

Chelariu-Raicu et al. designed a randomized phase-II study to evaluate the clinical efficacy and safety of a combination of EP-100 with PTX treatment in LHRH-R+ve expressed recurrent OVC patients. Overall response rate was quite similar in both the combination (35%) and the PTX treatment (33%) groups. However, liver metastases showed a significantly better response in combination (69%) compared to PTX alone (16%). A threefold increase in time to progression (TTP) rate was observed in patients who failed PTX monotherapy and switched to combination therapy, indicating PTX resensitization. A post-study investigation using a functional LHRH ligand assay (rather than a commercial antibody test) found that EP-100 was only beneficial to functionally LHRH-R+ve tumors [75].

#### Summary

Overall outcomes showed variable clinical activity. CBR includes complete, partial, or sustained stable disease for ≥8–12 weeks, providing a broader indication of disease control. The combination of EP-100 with PTX yielded overall response rates comparable to PTX monotherapy, indicating that the addition of EP-100 did not substantially increase clinical benefit at the tested doses. Thus, while CBR values suggest some ability to delay progression, durable tumor responses remained limited. In the EP-100 phase II study, patient selection was based on commercial immunohistochemistry (IHC) antibody assays for LHRH-R expression. However, functional ligand-binding tests showed that only functionally active receptors showed clinical benefit. This difference suggests that IHC misclassification and the inclusion of patients without functional receptor expression in the study. These early clinical findings collectively emphasize a further direction for LHRH-targeted therapy, suggesting that with better patient-selection strategies and more consistent biomarker integration, their true therapeutic potential may be more accurately defined.

### 6.3. Possible Challenges on LHRH-Targeted Agents

Overall, LHRH analogue conjugated anticancer drugs consistently demonstrate improved tumor selectivity, enhanced cellular uptake, and cytotoxic potency in the LHRH-R^+ve^ OVC model. Despite strong preclinical performance, translational gaps remain. Clinical trials have shown limited response in patients with platinum-resistant OVC. Treatment with the LHRH antagonist (cetrorelix) yielded irregular and transient responses in widely pretreated populations, showing biological effects but insufficient clinical stability. AEZS-108 demonstrated modest antitumor activity in Phase II studies, with partial responses and disease stabilization in LHRH-R^+ve^ patients. However, the subsequent Phase III trial in advanced endometrial cancer failed to show any improvement in overall survival, progression-free survival, or response rate compared with DOX alone. Because AEZS-108 did not outperform the standard treatment, its clinical development was discontinued [76]. Ligand stability and off-target effects represent a significant barrier. LHRH-derived peptides are susceptible to enzymatic degradation in plasma and peripheral tissues, reducing the fraction of intact conjugate that reaches the tumor [77,78,79,80]. We observed that most studies used D-Lys^6^ LHRH conjugates; however, this leads to premature drug release due to the instability of the ester linker. Vankadara et al. designed a novel peptide-drug-conjugate D-Cys^6^-LHRH vedotin by replacing the linker-toxin with vedotin (MC-VC-PABC-MMAE). D-Cys^6^-LHRH vedotin showed higher potency in LHRH-R^+ve^ OVCAR-3 cells, which was 113 times and 77 times more potent than AN-152 and DOX, respectively. It also exhibited 13/48-fold selectiv-ity against non-cancerous cells (H-6036/MRC-5). D-Cys^6^-LHRH vedotin was also active against SK-OV3, confirming the expression of LHRH-R in these cell lines [81].

Uncertainty regarding the receptor status of widely used OVC cell lines has become a significant source of variability in LHRH-targeting studies. Völker et al. found that SK-OV-3 cells lack high-affinity LHRH-R binding sites and do not express LHRH-R mRNA; thus, LHRH agonists and antagonists have no effect on their proliferation [38]. SK-OV-3 cells are commonly used as an LHRH-R^−ve^ control in most studies. Their responsiveness to the D-Cys^6^-LHRH vedotin conjugate indicates potential receptor expression, emphasizing inconsistencies in current receptor-assessment methods and the necessity for further investigation.

Tumor penetration and internalization dynamics of peptide-based drugs are intrinsically constrained in solid tumors. OVC often exhibits dense stroma, abnormal vasculature, and elevated interstitial pressure, all of which limit deep penetration of large peptide–drug conjugates compared with small molecules. Even when LHRH ligands bind efficiently at the tumor surface, internalization and intracellular drug release must be precisely coordinated with endosomal trafficking and linker cleavage; suboptimal timing or premature release can reduce effective intratumoral drug concentration.

The EP-100 was well-tolerated in early-phase studies but demonstrated minimal antitumor activity. Most patients experienced only temporary disease stabilization, and no objective tumor responses were recorded. In a subsequent randomized phase II trial, adding EP-100 to paclitaxel did not improve response rates or progression-free survival compared with paclitaxel alone [75].

Additionally, methodological issues likely contributed to underperformance. Many trials were undertaken in extensively pretreated, platinum-resistant patients with an inherent poor prognosis, limiting the potential to identify a further effect.

## 7. LHRH-Conjugated Nanosystem for Receptor-Mediated Cancer Targeting

**Figure 6:**
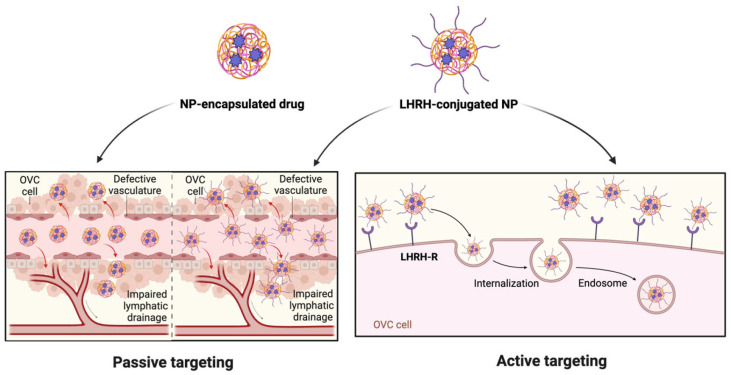
Targeting mechanism of NP-encapsulated drug vs. LHRH.

NPs have emerged as a powerful tool due to their unique physical and chemical characteristics, such as small size, high surface area-to-volume ratio, ease of surface modification, and ability to synthesize different and personalized NPs. In addition, their ability to encapsulate therapeutic agents and accumulate in tumor tissue through the enhanced permeability and retention (EPR) effect makes NPs an important drug delivery platform, improving drug solubility, enhancing bioavailability, and reducing systemic toxicity. In receptor-mediated targeting, NPs can be functionalized with ligands that bind specifically to receptors overexpressed on cancer cells. By conjugating LHRH peptides to nanosystems, it is possible to achieve precise delivery of anticancer drugs, thereby improving treatment efficacy and reducing off-target effects, making this a potent strategy in targeted OVC therapy (Figure 6) [82].

### 7.1. LHRH-Functionalized NPs for Drug Delivery

Several studies have explored LHRH-conjugated NPs for delivering different drugs, such as conventional chemotherapeutics, to ovarian tumors (Table 3). Pan et al. developed polycaprolactone NPs co-loaded with PTX and a near-infrared dye (IR780) with photothermal effect and engineered with an LHRH analogue to target drug-resistant. They demonstrated the therapeutic efficacy of combined therapy with PCL-LHRH/IR780-PTX in a drug-resistant OVC mouse model. Complete tumor eradication was observed in all mice treated with PCL-LHRH/IR780-PTX + NIR Laser irradiation, with no recurrence during the study. Mice treated with NPs without LHRH functionalization (PCL/IR780-PTX) and exposed to light showed an adequate response at the beginning of treatment, but also experienced a recurrence of the disease after a few days. This combination resulted in synergistic chemo-photothermal therapy that effectively inhibited drug-resistant ovarian growth in vivo [83].

Liposomes were widely studied as nanocarriers for several cytotoxic agents for the treatment of several cancers, including OVC. Qin et al. developed negatively charged liposomes incorporated with cholesterol hemisuccinate (CHS) for the delivery of docetaxel, achieving high entrapment efficiencies (92.07%) and drug loading (20.22%). Functionalization with LHRHa, via electrostatic adsorption, enhanced targeting specificity, with a 9-fold higher concentration of docetaxel in the tumor compared to injection of docetaxel alone in a SKOV3 xenograft mouse model. Furthermore, docetaxel delivery via LHRHa-liposomes resulted in reduced accumulation in off-target organs, particularly in the liver and spleen, supporting its potential for clinical translation [84]. Yuan et al. designed negatively charged liposomes, functionalized with LHRHa via electrostatic adsorption. Docetaxel was successfully encapsulated using a central composite design, achieving 93% encapsulation efficiency and 20% loading efficiency. In vitro studies demonstrated that LHRHa-modified liposomes exhibited significantly enhanced uptake by OVC cells compared to non-targeted controls, highlighting the importance of LHRH-R-mediated endocytosis in improving the delivery and therapeutic efficacy of docetaxel [85]. He et al. tested in vitro PEGylated liposomes modified with LHRH analogues for the delivery of mitoxantrone. The LHRH-mediated targeting significantly improved the cytotoxicity of cancer cells, probably due to receptor-mediated endocytosis of the liposomes and the intracellular drug release [86]. Ye et al. encapsulated a natural compound, Brucea javanica oil (BJOE), into LHRH-targeted liposomes (LHRH-BJOLs). In vitro studies demonstrated significantly stronger inhibitory effects on the proliferation of OVC cells compared to BJOE alone, non-functionalized BJOLs, and PBS as a control. In vivo treatment of OVC-bearing mice confirmed in vitro results with an increasing median survival time of 58.60 days (LHRHa-BJOLs), if compared with 42.40 days (PBS), 46.00 days (BJOE), and 52.00 days (BJOLs). In addition, tumor tissue analysis from mice treated with LHRHa-BJOLs showed an upregulation of Bax and caspase-3 and a downregulation of bcl-2 when compared to other groups (*p* < 0.05) [87].

Nanogels were designed to simultaneously carry multiple agents, promote prolonged circulation, enhance cellular uptake, and release the cargo only in response to intracellular environmental signals. Nukolova et al. synthesized LHRH-targeted nanogels for cisplatin delivery, which showed increased internalization via LHRH-receptor-mediated endocytosis in OVC cells. LHRH-nanogel/CDDP significantly reduced tumor size, reaching 75% tumor inhibition on the second day after treatment, compared to 50% of maximum inhibition achieved with free CDDP or nanogel/CDDP. Furthermore, free CDDP caused significant weight loss in mice, and nanogel/CDDP showed higher platinum accumulation in the liver and spleen compared to targeted delivery after LHRH-nanogels administration [88].

The FDA-approved PLGA (poly(lactic-co-glycolic acid) NPs, which are biocompatible and biodegradable nanocarriers, were also studied as a drug delivery system (DDS) for OVC treatment. Functionalized LHRH-PLGA NPs, encapsulating the chemotherapeutic agent CPT-11 (Irinotecan), were synthesized by Zhu et al. to improve the drug delivery to OVC cells that have developed resistance to platinum-based treatments (A2780/DDP cell lines). To further enhance tumor penetration and drug release, the NPs were used in combination with focused ultrasound to temporarily increase vascular permeability and improve NP accumulation within the tumor. In vitro experiments demonstrated that the LHRHa/CPT-11/PLGA NPs, when combined with focused ultrasound (FUS), significantly inhibited cell proliferation and induced S-phase cell cycle arrest. In vivo, these targeted NPs showed high specificity for LHRH-R-positive xenograft tumors derived from A2780/DDP cells, leading to a significant suppression of tumor growth when combined with focused ultrasound [89].

The LHRH peptide-conjugated NPs approach not only improves therapeutic efficacy but also reduces off-target toxicity. Taheri et al. reported that methotrexate (MTX) conjugated to human serum albumin NPs and further functionalized with LHRH peptides exhibited increased cytotoxicity and tumor inhibition in LHRH-R-positive OVC models, highlighting the advantages of ligand-mediated targeting [90]. Qi et al. introduced a dual-targeting strategy by designing NPs conjugated with both integrin αvβ3 and LHRH ligands. These NPs demonstrated significantly enhanced therapeutic outcomes due to increased cellular uptake through LHRH-R and integrin αvβ3 receptor-mediated endocytosis [91]. Wang et al. developed LHRH-containing biodegradable polymer micelles for enhanced intracellular drug delivery. These micelles promoted receptor-mediated endocytosis and increased accumulation in OVC cells [92]. Pu et al. conjugated PTX-loaded lipid microbubbles with LHRH, which were selectively destroyed by ultrasound for intraperitoneal OVC treatment. This tumor-site-specific drug delivery system provided controlled in situ drug release, resulting in significant tumor growth suppression in xenograft models [93]. Xu et al. developed FePt NPs that act as iron reservoirs for controlled Fe release. Iron release was found to be pH-dependent: at pH 4.8 (lysosomal conditions), 17% of iron was released after ~8 h, while at physiological pH 7.4, negligible release occurred. In contrast, Fe_3_O_4_ NPs used as controls showed minimal iron release under both conditions. ROS generation, assessed by DCFH-DA fluorescence, was significantly higher in A2780 cells incubated with FePt-LHRH NPs compared to HEK-293 cells, which express low LHRH-R levels. MTT viability assays showed enhanced cytotoxicity in A2780 cells treated with FePt-LHRH NPs compared to unmodified NPs [94]. In another study, Gao et al. developed PTX-loaded phase-transformation lipid NPs (PTX-anti-LHRHR-PTNPs) targeting OVC cells expressing LHRHRs. In vivo studies have demonstrated a slowing of tumor growth in murine ovarian models after PTX-anti-LHRHR-PTNPs injection. In addition, among all treatments, the PTX-anti-LHRHR-PTNPs + low-intensity focused ultrasound (LIFU) group exhibited the most potent antitumor effect, achieving a tumor inhibition rate (TIR) of 74.04% and the highest number of apoptotic cells. These results suggest that ultrasound-triggered drug release, combined with active targeting via LHRH-R, significantly enhances therapeutic efficacy [95].

Functionalizing NPs with an LHRH analogue offers enhanced tumor-specific uptake via receptor-mediated endocytosis, improving therapeutic efficiency while lowering systemic toxicity. Nanocarriers, such as liposomes, PLGA, and nanogel conjugated LHRH, have been shown to increase drug accumulation in tumors and improve treatment outcomes in both drug-sensitive and drug-resistant models [85,86,87,88]. These findings highlight the translational potential of NP-LHRH as a promising approach for OVC treatment.

### 7.2. LHRH-Functionalized NPs for Gene Therapy

The application of LHRH-conjugated nanosystems has been explored in the context of gene therapy for OVC, especially for targeted delivery of small interfering RNA (siRNA) with gene silencing effects (Table 4). Kim et al. developed polyelectrolyte complex (PEC) micelles coupled with LHRH analogue through PEG for the delivery of vascular endothelial growth factor (VEGF) siRNA in OVC. In vitro results showed an increase in cellular uptake of LHRH-functionalized micelles in A2780 cells (LHRH-R^+ve^) compared to non-targeted micelles. They also demonstrated efficient VEGF gene silencing via receptor-mediated endocytosis in OVC cells with minimal off-target effects [97].

Dendrimers are branched macromolecules that possess advantages such as a neutral surface for low cytotoxicity and highly organized compact NPs due to the existence of catanionic charges on their inner surface. Surface modification of dendrimers can enhance cellular uptake of siRNA. Shah et al. designed Polypropylenimine (PPI) dendrimers for the codelivery of siRNA targeted to CD44 mRNA and a chemotherapeutic agent (PTX). The overexpression of CD44 membrane receptor is not only responsible for tumor initiation and proliferation, but also for drug resistance and metastasis. The combination of gene silencing and chemotherapeutic delivery resulted in the suppression of CD44 mRNA and protein, as well as an efficient induction of cell death in metastatic OVC cells isolated from patients with malignant ascites [98].

Expanding on this concept, Schumann et al. developed a PEGylated PPI dendrimer conjugated LHRH for the delivery of DJ-1 siRNA [99]. The combination of DJ-1 knockdown with photodynamic therapy in a dendrimer nanoplatform functionalized with PEG and LHRH peptide (PPI-Pc) resulted in the complete eradication of OVC tumor from mice after a single dose of combined therapy. In contrast, tumors treated with photodynamic therapy (PDT) alone exhibited disease recurrence. In subsequent studies, the same nanosystem was used for combination therapy using DJ-1 siRNA and CDDP in both platinum-resistant and metastatic OVC. This system achieved over 80% knockdown efficiency at both mRNA and protein levels, significantly compromising the proliferation, viability, and migration of platinum-resistant cells. The therapeutic effect was further enhanced when combined with a low dose of cisplatin, particularly in platinum-resistant OVC cells with high basal DJ-1 expression. DJ-1 silencing inhibited the Akt pathway, reactivated p53 function, and increased oxidative stress, thereby sensitizing cancer cells to cisplatin. Notably, three treatment cycles combining DJ-1 siRNA with low-dose cisplatin resulted in complete tumor eradication in metastatic OVC, with no recurrence observed over 35 weeks. For both murine models, the results showed a higher therapeutic response with combined therapy than with siRNA-mediated DJ-1 knockdown and cisplatin treatment alone [100,101]. Dendrimers have also been utilized to deliver BCL-2-targeted siRNA. Patil et al. developed polyamidoamine (PAMAM) dendrimer functionalized LHRH for siRNA delivery targeting BCL2 mRNA in human OVC cells. The results showed LHRH-mediated targeting significantly enhanced siRNA internalization and gene silencing in cancer cells [102].

In another approach, Yu et al. developed a multifunctional Fe_3_O_4_-based NP system co-loaded with a cisplatin prodrug (Pt (IV)) and siRNA targeting EZH2 (siEZH2) and a surface functionalized PEG and LHRH for OVC targeting. The Pt (IV) prodrug was intracellularly reduced to active Pt (II), while LHRH functionalization significantly enhanced tumor-specific uptake. In vivo biodistribution studies showed minimal off-target accumulation, with prolonged retention of platinum at the tumor site and minimal toxicity. It is essential to emphasize that the co-delivery of siEZH2 overcame cisplatin resistance by targeting non-efflux mechanisms, resulting in the same level of cytotoxicity in both resistant and non-resistant tumor cells. The Fe_3_O_4_ core also provided MRI contrast capability, supporting its theranostic potential [103]. Another strategy explored by Chang et al. involved the use of functionalized LHRHa-microbubbles for non-viral gene delivery of wild-type p53 (wtp53) to OVC cells. They demonstrated that the targeted microbubbles significantly increased gene transfection efficiency and apoptosis in A2780/DDP cells expressing LHRH-R. The additional combination of ultrasound-targeted microbubble destruction (UTMD) technique showed an increase in wtp53 mRNA expression up to ~97% and cell apoptosis increased to nearly 40%. This non-invasive technique highlights the potential of physical stimuli to enhance the precision and efficacy of gene delivery [104].

A novel polymerization strategy was developed to synthesize azido-functionalized nano-capsules capable of ligand conjugation via copper-free click chemistry. By attaching tumor-targeting ligands such as LHRH peptide or anti-HER2 scFv, the nanocapsules were able to internalize into cancer cells. Using green fluorescent protein (GFP), they also confirmed the selective uptake in target cells. In addition, LHRH-conjugated nano-capsules successfully delivered functional p53 protein into cancer cells overexpressing LHRH-R, leading to reactivation of p53-dependent apoptosis [105]. Alatise et al. developed a multifunctional peptide platform capable of delivering siRNA selectively to OVC cells through LHRH-R targeting. Their system incorporates three functional domains: an LHRH-based targeting ligand, a cell-penetrating peptide, and an endosomal escape sequence. This design facilitated efficient internalization and cytosolic release of siRNA, resulting in effective gene silencing and reduced proliferation of cancer cells in vitro and in vivo [106]. Finally, Garbuzenko et al. highlighted the distinction between personalized and precision nanomedicine in the context of OVC. They addressed the inter-patient variability in response to chemotherapy, with an individual genetic and protein expression profile of each patient’s tumor. Based on this specific and individual genetic profile, they selected different siRNAs targeting each resistance mechanism, paired with a chemotherapeutic (PTX), and loaded into lipid NPs. The patient-derived OVC models were used to evaluate precision medicine (chemotherapy combined with a general set of siRNA) versus personalized medicine (chemotherapy combined with individually selected siRNA) versus standard treatment with PTX alone. Only the personalized treatment showed a significant reduction in tumor growth, confirming that the use of a specific siRNA to suppress the specific resistance mechanisms of each patient’s tumor is critical for improving therapeutic outcomes. Additionally, the LHRH-targeted system showed improved tumor localization and minimized off-target effects, thanks to dual passive and active targeting mechanisms [107].

The overall findings highlighted the emerging role of LHRH functionalized NPs in gene therapy for OVC treatment. These systems enable active targeting of siRNA genes such as CD-44, DJ-1, VEGRF, etc., with different nano platforms. These functionalized peptide nanosystems help to overcome drug resistance and tumor recurrence, thereby improving the therapeutic efficiency.

### 7.3. LHRH-Functionalized NPs with Theranostic Applications

The concept of theranostics combines both diagnostic and therapeutic agents on a single platform, allowing for the monitoring of accumulation and the real-time visualization of therapeutics at targeted tumor cells [108]. Many research studies on the nano-theranostic approach for cancer treatment have reported positive outcomes (Table 5), including a stable physiological environment and biodegradable and biocompatible properties [109]. LHRHa conjugated nano theranostic platform has been considered a promising approach in OVC treatment. Vishwasrao et al. developed magnetite-based nanoclusters loaded with cisplatin (MNCs) and conjugated with LHRHa and evaluated the imaging performance and therapeutic efficacy in cisplatin-sensitive (A2780-WT) and resistant (A2780-CisR) cells. Targeted MNCs showed significantly higher cellular uptake than non-targeted MNCs in both cell types, and the IC_50_ of targeted MNCs was lower than that of non-targeted ones. The increased uptake of targeted MNCs further improved the T2-weighted negative contrast in the cellular phantom, indicating a potential theranostic approach for OVC [110]. Taratula et al. designed a PPI G4 dendrimer nano theranostic system encapsulated with phthalocyanines (Pc) and modified with PEG-LHRH (Pc−PPIG4-PEG-LHRH). In LHRH-R+ A2780/AD cells, the LHRH nanosystem showed 3 times more cellular uptake than the non-targeted one, while in LHRH-R- SKOV-3 cells, the LHRH nanosystem showed minimal uptake, indicating receptor-specific targeting. LHRH nanosystem also exhibited a stronger PDT effect with reduced cell viability (IC_50_ 0.9 µg/mL). In vivo, tumor imaging revealed that the LHRh nanosystem efficiently accumulated in tumors and was capable of internalization into cancer cells [111]. In 2015, Taratula et al. developed another graphene-based nano LOGr-PC-PPIG4-PEG-LHRH theranostic system for targeted imaging and combinatorial phototherapy. The combination of photothermal therapy (PTT) and PDT increased OVC cell death, achieving 90–95% killing efficacy at low oxygen-graphene and Pc doses, indicating a synergistic effect. Precise tumor-specific fluorescence imaging has been observed in LHRH-NPs treated A2780/AD tumor-bearing mice after 12 h post-injection [112]. Lin et al. designed LHRH conjugated dual-responsive hyaluronic acid NPs. In vitro studies showed a higher tumor uptake of LHRH-NPs in OVCAR-3 cells than in normal 3T3 cells, and also obtained >80% cell viability inhibition after 72 h. LHRH-NPs showed effective tumor accumulation in an orthotopic ovarian tumor, with a reduction in tumor volume of ~30% after 20 days of treatment. LHRH-NPs were labeled with Cy 5.5 near-infrared (NIR) dye for in vivo and ex vivo tumor imaging. LHRH-NPs exhibited higher tumor accumulation than non-targeted NPs after 24 h post-injection, while ex vivo imaging showed minimal fluorescence in non-targeted organs [113].

LHRH-conjugated NPs have also been studied for OVC imaging, which could be a potential application for further nanotheranostic applications. Savla et al. fabricated LHRH-modified PEGylated manganese oxide NPs (LHRH-Mn_3_O_4_ NPs) for analyzing both imaging and therapy. LHRH-modified NPs showed preferential tumor accumulation in the subcutaneous OVC model, resulting in an increasing magnetic resonance imaging (MRI) signal intensity of ~22–3% [114]. Kumar et al. developed LHRH conjugated gold NPs (LHRH-AuNPs) to evaluate targeted imaging in OVC by using multi-energy spectral photon-counting computed tomography (MARS SPCCT). In vitro SPCCT imaging showed a higher accumulation of LHRH-AuNPs in SKOV3 OVC cells than non-conjugated NPs. Whereas in vivo SPCCT imaging revealed scattered accumulation of gold in the abdominal cavity, it is possible that gold was misidentified as calcium due to a high detection threshold and low payload. In vitro therapy showed potent biocompatibility, a slightly antiproliferative effect, and no significant toxicity [115]. Liu et al. functionalized LHRHa with ultrasmall gold NPs (Au-LHRHa) for dual-mode imaging-guided photothermal therapy of OVC. In vitro imaging showed that targeted SKOV3 cells had higher uptake of Au-LHRHa than non-targeted AuNDs. In vivo, CT imaging confirmed the higher contrast for Au-LHRHa than non-targeted AuNPs. A higher fluorescence intensity was found in the tumor than in the liver, kidney, and lungs. PTT results showed that Au-LHRHa with NIR significantly induced cell death and apoptosis, while in vivo photothermal therapy confirmed stronger tumor suppression than the control and non-targeted groups [116]. These findings suggest that LHRH functionalized with NPs can deliver both an imaging agent and a drug to the tumor site, working as a potential nanotheranostic platform.

A novel thermal decomposition method was used to develop Cobalt-doped iron oxide NPs with a unique cubical bipyramid shape, achieving exceptional magnetic hyperthermia performance (SAR ≈ 14,686 W/g Fe). Functionalized with LHRH for targeted delivery, these NPs demonstrated an efficient tumor accumulation and biocompatibility in vivo. At a low systemic dose, they rapidly reached therapeutic temperatures (>43 °C in 9 min), leading to effective suppression of tumor growth after a single 30-min hyperthermia session. This work highlights the promise of LHRH-targeted nanomaterials for treating deep-seated and metastatic tumors, although further safety evaluation is needed due to the cobalt content [96]. Magnetic NPs composed of magnetite (Fe_3_O_4_) core and a maghemite (γ-Fe_2_O_3_) shell were also used for systemic magnetic hyperthermia. Demessie et al. developed these NPs using a modified thermal decomposition method under a low nitrogen flow rate (10 mL/min), resulting in a high intrinsic loss power (ILP) of 47.5 nH·m^2^/kg. Indeed, they demonstrated a direct correlation between the shell thickness of NPs and heating efficiency, with the shell morphology that can be modified via the nitrogen flow rate. Using the theranostic design of these NPs (hyperthermia and MRI), the authors evaluated their accumulation in ES-2 subcutaneous tumor xenografts via T_2_-weighted imaging. Quantitative analysis of inductively coupled plasma mass spectrometry (ICP-MS) measurements of iron concentration confirmed that LHRH-targeted NPs achieved significantly greater tumor accumulation, with 2.4% ID/g compared to 1.1% ID/g for non-functionalized-NPs, 48 h after systemic injection of a low 4 mg/kg dose. Crucially, NPs showed high heating capacity when exposed to an alternating magnetic field (AMF, 420 kHz, 26.9 kA/m), reaching an intra-tumoral temperature of 50 °C and high therapeutic efficiency [117].

Nano-theranostics is a dual-functioning strategy. The findings so far demonstrated that LHRH conjugation significantly improves imaging ability, including MRI, CT, fluorescence, and photothermal signals, as well as improvements in vitro cytotoxicity and in vivo tumor suppression, even in drug-resistant models. Some studies found synergistic effects when phototherapy or ultrasound-triggered release was combined with LHRH-targeting. Overall, LHRH-functionalized theranostic NPs provide a potent, tumor-targeted, and multifunctional strategy for diagnosing and treating OVC.

### 7.4. Possible Challenges on LHRH-Conjugated Nanosystem

LHRH-conjugated nanosystems present several advantages that support their translational potential for ovarian cancer therapy.

Across different nanoplatforms, including liposomes, PLGA nanoparticles, nanogels, micelles, lipid carriers, and metallic nanosystems, LHRH functionalization consistently enhances tumor-specific uptake through receptor-mediated endocytosis. This results in higher intratumoral drug accumulation, improved therapeutic efficacy, and reduced systemic toxicity compared to non-targeted formulations.

Moreover, several studies demonstrate that LHRH targeting can overcome drug resistance, particularly when combined with other strategies such as photothermal therapy, ultrasound, or dual-receptor targeting [83,89,93,95,99,104,112,116]. The recurring trends of increased cytotoxicity in LHRH-R–positive ovarian cancer cells, lower drug accumulation in off-target organs (e.g., liver and spleen), and improved in vivo survival outcomes further underscore the importance of this targeting strategy.

Despite these promising results, several challenges persist in their clinical translational studies.

First, most studies are limited to early preclinical models, often using a single ovarian cancer cell line or xenograft with high LHRH-R expression, which may not reflect the heterogeneity of receptor levels in patients.

The stability of the ligand–nanocarrier linkage under physiological conditions also varies among platforms, particularly for systems relying on electrostatic adsorption rather than covalent attachment, potentially affecting targeting efficiency in vivo [84,85]. Additionally, the biodistribution and long-term safety of repeated administration remain insufficiently characterized, especially for metallic or hybrid nanoparticles that may accumulate in organs over time. Another challenge lies in the lack of direct comparative studies between different LHRH-functionalized nanocarriers, which limits the ability to determine which platforms offer the best balance of efficacy, biocompatibility, and manufacturability. Finally, large-scale, GMP-compatible production of ligand-modified nanosystems remains technically demanding, and regulatory pathways for targeted nanomedicines are still evolving. Addressing these issues will be essential to translate the strong preclinical performance of LHRH-conjugated nanosystems into clinically viable therapies for ovarian cancer.

## 8. Future Perspectives and Conclusions

In ovarian cancer, the use of peptide-conjugated delivery systems has gained significant attention, particularly those functionalized with LHRH or its analogues, due to the overexpression of LHRH-R on the tumor cells. This receptor overexpression allows a more efficient and selective targeting of the tumor region. A growing number of preclinical and clinical investigations are currently exploring LHRH-based peptide drug conjugates, suggesting that this approach is a promising therapeutic strategy for targeted treatment of OVC.

Additionally, in the current nanotechnology era, the functionalization of different nanosystems, such as liposomes, polymeric NPs, dendrimers, micelles, etc., with LHRH peptides/analogues has shown promising results. Indeed, these nanoplatforms engineered with LHRH demonstrated enhanced specificity for tumor cells through receptor-mediated targeting. This approach not only improves intracellular uptake and accumulation at the tumor site but also significantly reduces off-target toxicity. Furthermore, the integration of gene therapy components, such as siRNA, loaded into LHRH-targeted nanocarriers has opened new, promising, and personalized treatments in OVC.

Radiolabelling of LHRH peptides represents another promising but relatively unexplored research area, which could provide a theranostic approach to OVC. For example, Schottelius et al. (2008) [118] developed ^18^F- and ^68^Ga-labeled LHRH analogues using spacers such as aminohexanoic acid (Ahx) or β-alanine for the PET imaging of LHRH-R. Despite high receptor affinity and efficient internalization, in vivo tumor uptake was limited, underlining the need for further optimization of LHRH-targeted imaging agents [118]. Future research could focus on improving radiotracer stability, exploring alternative chelators or radionuclides, or combining radiolabelled LHRH peptides with NPs-based delivery systems to enhance tumor retention. Although this was the only identified study that specifically explored radiolabelled LHRHa in ovarian cancer, it highlights the need for further investigation and the development of tailored LHRH-based radiopharmaceuticals for ovarian cancer imaging and therapy.

Early-stage diagnosis of ovarian cancer remains a major clinical challenge. Although LHRH-targeted therapy has not yet been established as a standard treatment for recurrent OVC, numerous studies have demonstrated its potential to improve therapeutic precision and to reduce systemic toxicity. By enabling receptor-specific delivery of drugs, genes, or imaging agents, LHRH-based systems could significantly improve clinical outcomes, particularly in patients with LHRH-R-positive tumors. This targeted approach enhances treatment efficacy while minimizing systemic toxicity, supports personalized therapy based on receptor expression, and opens the door to combination strategies that may overcome drug resistance and improve long-term disease control.

In addition, the development of multifunctional nanoplatforms capable of combining targeted delivery, imaging, and controlled release represents a valuable tool for integrating diagnosis and therapy into a single system. Clinically, this approach offers significant advantages. For example, the same LHRH-functionalized nanosystem could first be used for diagnostic imaging by delivering a contrast or radioactive agent to confirm LHRH receptor overexpression in the tumor. Once validated, the same platform could then be loaded with therapeutic agents, allowing a translation from diagnosis to targeted treatment, thereby optimizing therapeutic precision and improving patient management.

A similar concept could also be applied to the use of the LHRH peptide alone, initially radiolabelled with diagnostic agents to evaluate receptor expression, and subsequently labeled with therapeutic radionuclides for treatment (theranostic approach). Although only a few studies have explored this strategy so far, the preliminary results suggest high potential. Given its ability to combine precise tumor characterization with targeted therapy in a single molecular platform, this approach represents a promising and emerging area of research that needs further investigation and could become a future hot topic in the management of ovarian cancer.

Future research directions should include ligand engineering to optimize LHRH analogues for improved stability, affinity, and internalization, as well as linker optimization to enable more efficient, tumor-responsive, and controlled release of therapeutic drugs. In addition, combining LHRH-mediated targeting with immunotherapy, or with potent innate immune activators such as STING agonists, represents a promising strategy to enhance antitumor immunity and overcome the immunologically “cold” nature of OVC. Finally, the design of advanced multifunctional and theranostic nanoplatforms capable of integrating imaging, targeted delivery, and immune modulation will be essential to guide personalized treatment and further improve patient outcomes.

Continued interdisciplinary research is therefore essential to translate these promising findings into clinically viable strategies for improved management of OVC.

## Figures and Tables

**Table 2:** Overview of clinical studies on LHRH-conjugated chemotherapeutics for OVC targeting therapy.

LHRHa/ Intervention	Patient (n)	Median Age/Range	Condition	Dosage	Treatment Duration	Response Rate%	Trial Phase	Trial Status	Ref.
Cetrorelix	17	58 (46–76)	Platinum-resistant OVC	10 mg/day subcutaneous (after initial 7-day dose-escalation)	Median four cycles (range 2–15)	18% PR (n = 3) 35% SD (n = 6)	Phase-II	Completed	[72]
AN-152	42	61 (37–77)	Platinum-resistant OVC	267 mg/m^2^ IV every 3 weeks	6–8 cycle (21 days each)	14.3% PR (n = 6) 38% SD (n = 16)	Phase-II	Completed	[73]
EP-100	8	59 (39–80)	Advanced solid tumors	2.6–11.7 mg/m^2^	≥16 weeks	25% SD (n = 2)	Phase-I	Completed	[74]
EP-100+PTX, PTX alone	44 (23 combo, 21 control)	60 (25–75) 68(43–91)	Recurrent OVC	EP-100 30 mg/m^2^ twice weekly + Paclitaxel 80 mg/m^2^ weekly	Median four cycles (up to 16)	35% in combo, 33% in control	Phase-II	Completed	[75]

**Table 3:** Summary of LHRH functionalized NPs for drug delivery in OVC treatment.

NPs Type; Physico-Chemical Properties	Conjugated LHRH; Modification Method (Peptide/NP Ratio)	Therapeutic/ Diagnostic Agent Delivery	In Vitro Model (Cell Line, Assay, EC_50_)	In Vivo Model (Cell Type); Injection Route of Pharma	In Vivo Outcome (Median Survival, Tumor Reduction Size)	Key Findings	Ref.
Poly-ε-caprolactone (PCL) NPs; 140–160 nm PCL/IR780-PTX; −20 to −30 mV	LHRHa; covalent conjugation via Schiff’s base reaction	Paclitaxel (PTX); IR780 fluorescent agent	Cytotoxicity assay on paclitaxel resistant cell line SKOV3-TR30 (ST30 cells), IC_50_ of 1285 nM	BALB/c nude mice bearing ST30 xenograft; intravenous injection	Tumor-growth inhibition ratio of 100% for combination therapy (PCL-LHRH/IR780-PTX + Light)	Complete regression of tumor and no recurrence of disease in vivo after treatment with a combination of chemo-photothermal therapy plus functionalized PCL.	[83]
Liposomes; 335 ± 15 nm, −26.4 ± 2.1 mV	LHRHa; electrostatic adsorption (1:2)	Docetaxel (CAS 114977-28-5)	Limit of detection (LOD): 0.12 μg/mL; limit of quantification (LOQ) 0.4 ng	Athymic (nu/nu) mice with SKOV 3 cells xenograft; intravenous injection.	Only the biodistribution study	Nine-fold increase in tumor accumulation and reduced off-target distribution of docetaxel in vivo after treatment with LHRHa-targeted liposomal delivery system.	[84]
Liposomes; 342 ± 21 nm; −30 to −35 mV	LHRHa; electrostatic adsorption (1:1, 1:2, 1:5, 1:10)	Docetaxel	Cell uptake study with SKOV3 cells (highest value with a ratio of 1:2 LHRHa/liposomes after 2 h of incubation)	NA	NA	Enhanced cellular uptake achieved in vitro using LHRHa-targeted liposomes loaded with docetaxel for OVC therapy.	[85]
PEGylated liposomes; 120–145 nm; −18 to −25 mV	Gonadorelin LHRHa (Pyr-His-Trp-Ser-Tyr-Gly-Leu-Arg-Pro-Gly-NH2); covalent binding (2:1)	Mitoxantrone hydrochloride	Cytotoxicity assay on SKOV3 cells (IC_50_ of 0.578 μg/mL)	NA	NA	NPs facilitated the specific delivery of the drug to LHRH-R overexpressing tumor cells and efficiently inhibited tumor growth.	[86]
Liposomes; 155.1 ± 14.5 nm; −24.1 ± 0.54 mV	LHRHa; Biotin-avidin conjugation (biotinylated LHRHa peptide + avidinylating NPs)	Brucea javanica oil (BJOLs)	Cell viability assay on A2780/DDP cells [inhibitory rates of (37.66 ± 1.73)%, (51.26 ± 3.46)%, and (65.45 ± 4.42)% at 24, 48, and 72 h after treatment, respectively].	Nude athymic (nu/nu) mice bearing A2780/DDP xenograft; intravenous injection.	Median survival: 58.60 ± 1.03 days.	BJOLs and LHRHa-BJOLs groups of mice had significantly longer survival times than PBS and BJOE groups. In particular, LHRHa-BJOLs exhibited the longest survival time.	[87]
Nanogel (Poly (ethylene glycol)170-b-poly (methacrylic acid)180 (PEG-b-PMA) diblock copolymer); 139 ± 4 nm and −22.1 ± 1,7 mV (in water, pH7.4),128 ± 4 nm and −6.8 ± 1.1 mV (in PBS, pH 7.4)	(D-Lys6)-LHRHa; covalent conjugation with EDC/NHs chemistry (1:1)	Cisplatin (_C_DDP); FITC	Cytotoxicity assay on A2780 cells (IC_50_ of 9.24 ± 0.9 μg/mL)	Nude athymic (nu/nu) mice bearing A2780 xenograft; intravenous injection.	Tumor inhibition: ~75% from day 2, sustained for 20 days; significantly reduced tumor volume compared to other groups; longest survival; no systemic toxicity	LHRH-nanogels/_C_DDP were more effective and less toxic than equimolar doses of free _C_DDP or nanogels/_C_DDP in the treatment of LHRH-R-positive cancers.	[88]
Poly lactic-co-glycolic acid (PLGA) NPs; 637.4 ± 57.0 nm; −6.48 ± 6.71	LHRHa; covalent conjugation with EDC and INHS chemistry	Irinotecan (CPT-11) + ultrasound (US); FITC	Cytotoxicity assay on A2780/DDP cells (IC_50_ of 0.2 mg/mL at 72 h)	BALB/c nude mice bearing A2780/DDP xenograft; intravenous injection	Tumor-inhibition rate 73.5% (LHRH-a/CPT-11/PLGA with US)	Superior efficacy of the LHRH-a/CPT-11/PLGA with US treatment over other tested groups, including various controls and CPT-11-based treatments without targeted microspheres or ultrasound	[89]
Human serum albumin NPs (HSA); 120–138 nm; −10 to −12 mV	LHRHa; covalent conjugation with EDC chemistry (2, 5, and 10 mg of LHRH added to MTX-HSA NPs)	Methotrexate (MTX)	Cytotoxicity assay on T47D cells (IC_50_ of 5.82 ± 1.08 nM)	NA	NA	Active targeting with LHRH-MTX-HSA NPs significantly increased the anti-tumoral activity of MTX at low concentrations in comparison to non-targeted MTX-HSA NPs.	[90]
Liposomes; 108.52 ± 1.63 nm; −22.07 ± 1.56 mV	LHRHa/RGD co-modified NPs; thioether bond with the Mal functional group at the N-terminus of the liposomes-Mal chain	Paclitaxel (PTX)	Cytotoxicity assay on A2780/DDP cells (IC_50_ of 0.19 µg/mL)	BALB/c nude mice bearing A2780/DDP xenograft; intravenous injection	Tumor weight reduction: LHRHa-RGD-LP-PTX group (0.29 ± 0.07 g) vs. control (1.90 ± 0.48 g)	LHRHa and RGD co-modified liposomes enhanced the in vivo anti-tumor efficacy against LHRH-R-positive OVC	[91]
Micelles composed by triblock copolymers (poly (ethylene oxide)-block-poly (allyl glycidyl ether)-block-poly(DL-lactide) (mPEG-b-PAGE-b-PLA); 15–40 nm	LHRHa; covalent conjugation with EDC/NHs chemistry (0.2:1)	Doxorubicin (DOX); FITC	Cellular uptake study on SKOV3 cells	Nude athymic (nu/nu) mice bearing SKOV3 cells xenograft; intravenous injection.	Only the biodistribution study	LHRH-functionalized micelles can be endocytosed more efficiently by LHRH-R^+ve^ cells than by LHRH-R^−ve^ cells. More LHRH-containing micelles were accumulated in the tumor site than LHRH-free micelles at 24 h post administration.	[92]
Lipid microbubbles (TPLMBs); 1.8 ± 0.2 μm; −9.6 ± 3.2 mV	LHRHa (pGlu-His-Trp-Ser-Tyr-D-leu-leu-Arg-Pro-NH2); Biotin-avidin conjugation (biotinylated LHRHa peptide + avidinylating NPs)	Paclitaxel (PTX) + ultrasound (US)	Quantitative assessment of apoptosis on ex vivo tumor (A2780/DDP cells). Strongest tumor apoptosis with TPLMBs + US treatment: (AI 55.94 ± 8.94%)	BALB/c nude mice bearing A2780/DDP xenograft; intravenous injection.	Median survival with TPLMBs + US treatment: +52% vs. control.	Ultrasound-mediated destruction of drug-loaded microbubbles after intraperitoneal administration led to a superior therapeutic outcome in comparison with other treatment options	[93]
Magnetic NPs (Fe_40_Pt_60_); 60–80 nm	LHRH peptide [Gln-His-Trp-Ser-Tyr-DLys(DCys)-Leu-Arg-Pro-NHEt]; covalent binding with EDC + sulfo-NHS chemistry [FePt-COOH NPs (10 mg)+ 0.4 mg of EDC (2 mM), 1.1 mg of sulfo-NHS (5 mM)+ 0.5 mg of LHRH peptide]	Fe (CO)_5_ used as a therapeutic agent	MTT viability assay with A2780 cells (IC_50_ of 1.25 µg Fe/mL)	NA	NA	FePt NPs act as a stable Fe reservoir at physiological pH but release Fe under acidic lysosomal conditions, making them promising agents for targeted ROS-mediated cancer therapy.	[94]
Lipid NPs; 508 ± 11.26 nm; −30.53 ± 6.34 mV	LHRH-R mAb Biotin-avidin conjugation (biotinylated LHRHR mAb + avidinylating NPs)	Paclitaxel (PTX) + low-intensity focused ultrasound (LIFU)	Cytotoxicity assay on A2780 cells and OVCAR-3 cells (the survival rate of cells gradually decreased with the increasing concentration of PTX)	Nude athymic (nu/nu) mice bearing OVCAR-3 cells; intravenous injection.	Tumor inhibition rate of ~80% with PTX-anti- LHRHR-NPs + LIFU	The combination therapy of PTX-anti-LHRHR-PTNPs + low-intensity focused ultrasound resulted in enhanced drug release and high therapeutic outcomes.	[95]
Cobalt-doped iron oxide NPs (Co-IONPs); 14.5 ± 2.5 nm; +12.5 ± 0.61 mV	LHRHa; covalent conjugation via Michael addition (maleimide-thiol reaction)	Magnetite and maghemite phases for magnetic hyperthermia; NIR fluorescence dye SiNc	Cell viability assay (reduction of 35% viability)	BALB/c nude mice bearing ES-2 xenograft; intravenous injection	100% inhibition of tumor growth with combination therapy	Complete inhibition of tumor growth in vivo after a single magnetic hyperthermia session using LHRH-targeted cubical bipyramidal Co-doped iron oxide NPs.	[96]

**Table 4:** Summary of LHRH functionalized NPs for gene therapy in OVC.

NPs Type; Physico-chemical Properties	Conjugated LHRH; Modification Method (Peptide/NP Ratio)	Therapeutic/ Diagnostic Agent Delivery	In Vitro Model (Cell Line, Assay, EC_50_)	In Vivo Model (Cell Type); Injection Route of Pharma	In Vivo Outcome (Median Survival, Tumor Reduction Size)	Key Findings	Ref.
Polyelectrolyte complex (PEC) micelles; 150 nm	LHRHa (Gln-His-Trp-Ser-Tyr- DLys-Leu-Arg-Pro); covalent conjugation with EDC and NHs chemistry	VEGF siRNA; Cy3 fluorescent agent	In vitro VEGF gene suppression assay on SK-OV-3 cells (gene silencing of 70.4 ± 9.4%) and on A2780 cells (gene silencing of 63.2 ± 4.0%)	NA	NA	VEGF siRNA-PEG-LHRH/PEC micelles specifically inhibited VEGF expression in cancer cells in an LHRH-R-specific manner	[97]
Polypropylenimine (PPI)dendrimer; 100–200 nm, +1.10 ± 1.54 mV	LHRHa [Gln-His-Trp-Ser-Tyr-DLys(D-Cys)-Leu-Arg-Pro)]; covalent conjugation with EDC and NHs chemistry (2:1)	Paclitaxel + CD44 siRNA; NuLight DY-547 fluorophores	Cytotoxicity assay on cancer cells obtained from the peritoneum area of patients with OVC (viability of ascitic cells decreased almost 10-fold when compared with control cells)	Nude athymic (nu/nu) mice bearing human ascitic xenograft; intravenous injection	Combination therapy led to the almost complete shrinkage of the tumor within the 28-day study period	High therapeutic potential for a combinatorial approach using siRNA against CD44 and cytotoxic agents delivered via PPI dendrimer.	[98]
PPIG4 dendrimer; 147.5 ± 0.2 nm; +11.9 ± 0.2 mV;	LHRHa ((Gln-His-Trp-Ser-Tyr-DLys(DCys)-Leu-Arg-Pro-NH-Et), w); covalent conjugation with thiol–maleimide reaction	DJ-1 siRNA + photodynamic therapy (PDT); phthalocyanine	Viability assay on ES2 and A2780/AD cells (therapeutic efficacy of the combinatorial approach improved by 6–20% compared to PDT alone)	Nude athymic (nu/nu) mice bearing A2780/AD cells	Tumors treated with combinatorial therapies were almost eradicated from the mice on the 15th day after the treatment	LHRH-targeted nanoplatform delivering DJ-1 siRNA in combination with phthalocyanine-based PDT enhanced ROS generation, overcame oxidative stress resistance, and achieved complete tumor eradication in cisplatin-resistant cells with high DJ-1 expression.	[99]
PPIG4 dendrimer 147.8 ± 11.0 nm; +6.44 ± 2.14	LHRHa (Gln-His-Trp-Ser-Tyr-DLys(DCys)-Leu-Arg-Pro-NH-Et); covalent conjugation with thiol–maleimide reaction (5:1)	DJ-1 siRNA + cisplatin (_C_DDP)	Cell viability assay on A2780/CDDP, ES2, and IGROV-1 cells (IC_50_: 23.6 μM, 3.8 μM, and 1.9 μM, respectively)	NA	NA	LHRH-targeted nanoplatform delivering DJ-1 siRNA in combination with _C_DDP enhanced apoptosis, reduced proliferation, and increased ROS levels in cisplatin-resistant OVC	[100]
PPIG4 dendrimer; 145.2 ± 9.1 nm; +7.7 ± 1.6 mV	LHRHa; covalent conjugation with thiol–maleimide reaction (1.6 μmoles of the LHRH: 50 nanomoles of siRNA)	DJ-1 siRNA + cisplatin (_C_DDP)	Luciferase-expressing ES-2 (ES-2-luc) cells	Nude athymic (nu/nu) mice bearing ES-2 xenograft; intraperitoneal injection	Mice treated with combinatorial therapies (median survival time >35 vs. 3 weeks for the control group). Reduction in the hazard ratio by 89.7% compared to saline-treated animals	The combination of DJ-1-targeted siRNA with low-dose _C_DDP using an LHRH-functionalized dendrimer nanoplatform enhanced cytotoxic efficacy, overcame _C_DDP resistance, and achieved complete tumor eradication in vivo.	[101]
PAMAM dendrimer 150 nm; +0.11 ± 0.88 mV	LHRHa [Lys6-des-Gly10-Pro9-ethylamide (Gln-His-Trp-Ser-Tyr-d-Lys-Leu-Arg-Pro-NH-Et)]; covalent conjugation via ester bond	BCL2 siRNA; FITC	MTT assay on A2780 cells with plane LHRH-PAMAM (5–10% of cells with high concentrations up to 12.5 μM)	NA	NA	Dendrimers targeting the plasma membrane of cancer cells significantly enhanced siRNA delivery and gene silencing.	[102]
Fe_3_O_4_ NPs 8–10 nm; +22.9 ± 0.5 mV	LHRHa [(Glp-HWSY(D-K)LRPG-NH2)]; covalent conjugation with EDC and NHs chemistry	EZH2 siRNA + platinum prodrug; Fe3O4 as an MRI contrast agent	MTT assay on A2780 cells and A2780/DDP (IC_50_ of 21.9 ± 5.3 μM and 55.6 ± 9.6 μM, respectively)	BALB/c nude mice bearing A2780/DDP xenograft; intravenous injection	Significant reduction in tumor volume	Overcoming _C_DDP resistance and achieving targeted tumor accumulation in vivo using PEG-LHRH-functionalized Fe_3_O_4_ NPs co-delivering Pt (IV) and siEZH2.	[103]
Lipid microbubbles 2527.6 ± 496.4 nm	LHRHa (D-Leu6-des-Gly10-Pro9-ehtylamine); Biotin-avidin conjugation (biotinylated LHRHR peptide + avidinylating NPs)	pEGFPN1-wtp53 plasmid; ultrasound (US)	Cell apoptosis assay on A2780/DDP cells with combinatorial approach (39.67 ± 5.95%)	NA	NA	Enhanced gene transfection and apoptosis in OVC cells after ultrasound destruction of LHRHa-microbubbles delivering wtp53.	[104]
Protein nanocapsules 9.1 ± 0.8 nm; −0.9 mV	LHRHa (Glp-His-Trp-Ser-Tyr-D-Lys-Leu-Arg-Pro-NHEt); covalent conjugation with amide linkage	Recombinant p53 protein	MTS assay on MDA-MB-231 cells (EC50 of 300 nM at 48 h)	NA	NA	LHRH-targeted nanocapsules enabled functional intracellular delivery of p53 and triggered apoptosis in OVC cells overexpressing LHRH-R.	[105]

**Table 5:** Summary of LHRH functionalized nanotheranostic systems in OVC.

NPs Type; Physico-Chemical Properties	Conjugated LHRH; Modification Method (Peptide/NP Ratio)	Therapeutic/ Diagnostic Agent Delivery	In Vitro Model (Cell Line, Assay, EC_50_)	In Vivo Model (Cell Type); Injection Route of Pharma	In Vivo Outcome (MEDIAN Survival, Tumor Reduction Size)	Key Findings	Ref.
Magnetic nanoclusters ~72.2 nm; −35.4 mV	LHRHa [d-Lys-6-LHRH (Glp–His–Trp–Ser–Tyr– DLys–Leu–Arg–Pro–Gly]; covalent conjugation with EDC and NHs chemistry	cisplatin (_C_DDP); Superparamagnetic iron oxide NPs as an MRI contrast agent	Cytotoxicity assay on A2780-WT and A2780-CisR cells (IC_50_ values at 24 h were 3.2 µM and 18.3 µM, respectively.)	NA	NA	Conjugation with LHRHa enhanced cellular uptake, improved cytotoxicity, and exhibited potential as an MRI contrast agent.	[110]
PPI G4 dendrimer NPs 62.3± 0.1; +24.4 mV	LHRHa [Lys6-des-Gly10-Pro9-ethylamide (Gln-His-Trp-Ser-Tyr-DLys(DCys)-Leu-Arg-Pro-NH-Et]; covalent conjugation with amide linkage (1:1)	Phthalocyanine for imaging and photodynamic therapy	Calcein AM cell viability assay on A2780-AD and SKOV3 cells (cell viability decreased by 78% after 15 min of light irradiation)	Nude athymic (nu/nu) mice bearing A2780-AD xenograft; intravenous injection	Only the biodistribution study	Higher tumor uptake, stronger photodynamic therapy upon 670 nm laser irradiation, and effective in vivo tumor imaging	[111]
Lowoxy graphene-dendrimer NPs ~78.3 ± 9.5 nm; +6.3 mV	LHRHa (Lys6–des-Gly10–Pro9-ethylamide [Gln–His–Trp–Ser–Tyr–DLys(DCys)–Leu–Arg–Pro–NH–Et]); covalent conjugation with thiol–maleimide reaction (1:1)	Phthalocyanine for imaging and photodynamic therapy	Calcein AM cell viability assay on A2780-AD cells (15 min of light irradiation resulted in only 5% of the cells surviving)	Nude athymic (nu/nu) mice bearing A2780-AD xenograft; intravenous injection	Only the biodistribution study	A combination of PTT and PDT resulted in 90–95% cell death and stronger tumor-targeted NIR imaging.	[112]
Self-assembling HA NPs 229 ± 5.6 nm	LHRHa (pyroGlu-His-Trp-Ser-Tyr-Gly-Leu-Arg-Pro-Gly-NH2); covalent conjugation with EDC and NHs chemistry (0.1:1)	Doxorubicin (DOX); Cy5 for fluorescent imaging	MTS assay on OVCAR-3 cells (inhibition of proliferation to less than 40% after 48 h)	Nude athymic (nu/nu) mice bearing OVCAR-3 xenograft; intravenous injection	ROI volume of the LHRH-NPs-treated group decreased to almost 30% of original size compared to that at the beginning of therapy	LHRH-NPs demonstrated enhanced cellular uptake with cytotoxicity in OVC cells, while exhibiting minimal cytotoxicity in normal cells, and strong tumor imaging with Cy5.	[113]
PEGylated Mn_3_O_4_ NPs 26.68 ± 3.49	LHRHa [Gln-His-Trp-Ser-Tyr-D-Lys(D-Cys)-Leu-Arg-Pro-NHEt]; covalent conjugation with EDC and NHs chemistry	Vemurafenib; Mn_3_O_4_ as MRI contrast agent	MTT assay on A2780 cells (IC_50_ 15 mcg/mL)	Nude athymic (nu/nu) mice bearing A2780 xenograft; intraperitoneal injection	Only the biodistribution study	Selective tumor accumulation with an increase of 22.3% of MRI signal intensity	[114]
AuNPs 26.40 nm; −8.78 mv	LHRHa (Ac-Gln-H is-Trp-Ser-Tyr-Gly-Leu-Arg-Pro-Gly-Lys-N Hz trifluoroacetate salt); covalent conjugation with EDC and NHs chemistry	Gold material for spectral photon-counting CT imaging	Apoptotic assay on SKOV3 and OVCAR5 cells (number of necrotic cells 1.18% and 4.39%, respectively)	C57/BL6 mice bearing syngeneic ovarian cancer (ID-8*Trp*53 wild-type cells); intraperitoneal injection	NA	LHRH-AuNPs showed significantly higher accumulation than AuNPs. Increasing LHRH per particle, they also improved the uptake without cytotoxicity.	[115]
Ultra-small AuNPs 3.2 nm; −21.6 mV	LHRHa (GRHa); covalent conjugation with EDC and NHs chemistry	Gold material for CT imaging; photothermal therapy	Cell migration assay on SKOV3 cells [lowest migration (31.86 ± 3.22%) compared to control (88.58 ± 2.80%)] with the combinatorial approach	BALB/c mice bearing syngeneic ovarian cancer (ID8 cells); intravenous injection	Mice with the combinatorial approach (Au-GRHa + NIR group) achieved the best tumor suppression effect	Au-GRHa enabled FL/CT dual-mode imaging, showed >67% targeted uptake in SKOV3 cells, and achieved significant tumor suppression via photothermal therapy.	[116]

## Data Availability

No new data were created or analyzed in this study. Data sharing is not applicable to this article.

## Abbreviations

NPs	Nanoparticles
LHRH	Luteinizing hormone-releasing hormone
GnRH	Gonadotropin-releasing hormone
LHRH-R	LHRH-Receptors
OVC	Ovarian cancer
PTX	Paclitaxel
LHRHa	LHRH analogues
GPCR	G-protein coupled receptor
PLC	Phospholipase C
EDC	1-ethyl-3-(3-dimethylaminopropyl)carbodiimide hydrochloride
NHS	N-hydroxysuccinimide
LHRH-R^+ve^	LHRH-R-positive
LHRH-R^−ve^	LHRH-R-negative
DOX	Doxorubicin
AN-152/AEZS-108	DOX-[D-Lys^6^]-LHRH
2DG	2-deoxy-D-glucose
AN-201	2-pyrrolino-DOX
AN-207	2-pyrrolino-DOX-[D-Lys^6^]-LHRH
AN-238	Somatostatin analogues
CPT	Camptothecin
PEG	Poly(ethylene glycol)
SD	Stable disease
EPR	Enhanced permeability and retention
CHS	Cholesterol hemisuccinate
BJOE	Brucea javanica oil
PLGA	Poly(lactic-co-glycolic acid
CDDP	Cisplatin (cis-diamminedichloroplatinum(II)
MTX	Methotrexate
siRNA	Small interfering RNA
VEGF	Vascular endothelial growth factor
PPI	Polypropylenimine
PAMAM	Polyamidoamine
PEC	Polyelectrolyte complex
MNCs	Magnetite-based nanoclusters loaded with cisplatin
Pc	Phthalocyanines
PDT	Photodynamic therapy
PTT	Photothermal therapy
CT	Computed tomography
MARS SPCCT	Multi-energy spectral photon-counting computed tomography
NIR	Near-infrared
MRI	Magnetic resonance imaging
MNPs	Superparamagnetic iron oxide NPs

## References

[1] Nayak P., Bentivoglio V., Varani M., Signore A. (2023). Three-Dimensional In Vitro Tumor Spheroid Models for Evaluation of Anticancer Therapy: Recent Updates. Cancers.

[2] Guo L., Wang J., Li N., Cui J., Su Y. (2023). Peptides for Diagnosis and Treatment of Ovarian Cancer. Front. Oncol..

[3] Global Cancer Observatory (GLOBOCAN) (2022). Globocan Factsheets. https://gco.iarc.who.int/media/globocan/factsheets/populations/900-world-fact-sheet.pdf.

[4] Goff B.A., Mandel L.S., Drescher C.W., Urban N., Gough S., Schurman K.M., Patras J., Mahony B.S., Andersen M.R. (2007). Development of an Ovarian Cancer Symptom Index. Cancer.

[5] Kim M., Lee Y.J., Seon K.E., Kim S., Lee C., Park H., Choi M.C., Lee J.-Y. (2025). Morbidity and Mortality Outcomes After Cytoreductive Surgery with Hyperthermic Intraperitoneal Chemotherapy for Treatment of Ovarian Cancer. J. Clin. Med..

[6] Baradács I., Teutsch B., Vincze Á., Hegyi P., Szabó B., Nyirády P., Ács N., Melczer Z., Bánhidy F., Lintner B. (2025). Efficacy and Safety of Combination Therapy with PARP Inhibitors and Anti-Angiogenic Agents in Ovarian Cancer: A Systematic Review and Meta-Analysis. J. Clin. Med..

[7] Hayek J., An A., Wolf J., Lamiman K., Kim M., Knochenhauer H., Goncalves N., Alagkiozidis I. (2025). Overall Survival Following Interval Complete Gross Resection of Advanced Ovarian Cancer via Laparoscopy Versus Open Surgery: An Analysis of the National Cancer Database. J. Clin. Med..

[8] Ledermann J.A., Raja F.A., Fotopoulou C., Gonzalez-Martin A., Colombo N., Sessa C. (2018). Corrections to “Newly Diagnosed and Relapsed Epithelial Ovarian Carcinoma: ESMO Clinical Practice Guidelines for Diagnosis, Treatment and Follow-Up”. Ann. Oncol..

[9] Zafar A., Khatoon S., Khan M.J., Abu J., Naeem A. (2025). Advancements and Limitations in Traditional Anti-Cancer Therapies: A Comprehensive Review of Surgery, Chemotherapy, Radiation Therapy, and Hormonal Therapy. Discov. Oncol..

[10] Li X., Taratula O., Taratula O., Schumann C., Minko T. (2017). LHRH-Targeted Drug Delivery Systems for Cancer Therapy. Mini-Rev. Med. Chem..

[11] Kunjiappan S., Panneerselvam T., Govindaraj S., Parasuraman P., Baskararaj S., Sankaranarayanan M., Arunachalam S., Babkiewicz E., Jeyakumar A., Lakshmanan M. (2020). Design, In Silico Modelling, and Functionality Theory of Novel Folate Receptor Targeted Rutin Encapsulated Folic Acid Conjugated Keratin Nanoparticles for Effective Cancer Treatment. Anticancer Agents Med. Chem..

[12] Gao H., Yang Z., Zhang S., Cao S., Shen S., Pang Z., Jiang X. (2013). Ligand Modified Nanoparticles Increases Cell Uptake, Alters Endocytosis and Elevates Glioma Distribution and Internalization. Sci. Rep..

[13] Prajapati A., Rangra S., Patil R., Desai N., Jyothi V.G.S.S., Salave S., Amate P., Benival D., Kommineni N. (2024). Receptor-Targeted Nanomedicine for Cancer Therapy. Receptors.

[14] Worm D.J., Els-Heindl S., Beck-Sickinger A.G. (2020). Targeting of Peptide-binding Receptors on Cancer Cells with Peptide-drug Conjugates. Pept. Sci..

[15] Varani M., Campagna G., Bentivoglio V., Serafinelli M., Martini M.L., Galli F., Signore A. (2021). Synthesis and Biodistribution of 99mTc-Labeled PLGA Nanoparticles by Microfluidic Technique. Pharmaceutics.

[16] Lastoria S., Rodari M., Sansovini M., Baldari S., D’Agostini A., Cervino A.R., Filice A., Salgarello M., Perotti G., Nieri A. (2024). Lutetium [177Lu]-DOTA-TATE in Gastroenteropancreatic-Neuroendocrine Tumours: Rationale, Design and Baseline Characteristics of the Italian Prospective Observational (REAL-LU) Study. Eur. J. Nucl. Med. Mol. Imaging.

[17] Varani M., Bentivoglio V., Campagna G., Nayak P., Lauri C. (2024). Evaluation of a New Avidin Chase in Murine Models Pre-Treated with Two 111In Labelled Biotin Analogues. Int. J. Appl. Biol. Pharm..

[18] Giorgio A., Varani M., Lauri C., Bentivoglio V., Nayak P. (2025). Radiolabeled LHRH and FSH Analogues as Cancer Theranostic Agents: A Systematic Review. J. Clin. Med..

[19] Bentivoglio V., D’Ippolito E., Nayak P., Giorgio A., Lauri C. (2025). Bispecific Radioligands (BRLs): Two Is Better Than One. J. Clin. Med..

[20] Kunjiappan S., Pavadai P., Vellaichamy S., Ram Kumar Pandian S., Ravishankar V., Palanisamy P., Govindaraj S., Srinivasan G., Premanand A., Sankaranarayanan M. (2021). Surface Receptor-mediated Targeted Drug Delivery Systems for Enhanced Cancer Treatment: A State-of-the-art Review. Drug Dev. Res..

[21] Lappano R., Maggiolini M. (2012). GPCRs and cancer. Acta Pharmacol. Sin..

[22] Obayemi J.D., Salifu A.A., Eluu S.C., Uzonwanne V.O., Jusu S.M., Nwazojie C.C., Onyekanne C.E., Ojelabi O., Payne L., Moore C.M. (2020). LHRH-Conjugated Drugs as Targeted Therapeutic Agents for the Specific Targeting and Localized Treatment of Triple Negative Breast Cancer. Sci. Rep..

[23] Coelingh Bennink H.J.T., Roos E.P.M., van Moorselaar R.J.A., van Melick H.H.E., Somford D.M., Roeleveld T.A., de Haan T.D., Reisman Y., Schultz I.J., Krijgh J. (2024). Estetrol Inhibits the Prostate Cancer Tumor Stimulators FSH and IGF-1. J. Clin. Med..

[24] Zhang Q., Madden N.E., Wong A.S., Chow B.K., Lee L.T. (2017). The Role of Endocrine G Protein-Coupled Receptors in Ovarian Cancer Progression. Front. Endocrinol..

[25] McArdle C., Franklin J., Green L., Hislop J. (2002). Signalling, Cycling and Desensitisation of Gonadotrophin-Releasing Hormone Receptors. J. Endocrinol..

[26] Krsmanovic L.Z., Martinez-Fuentes A.J., Arora K.K., Mores N., Tomić M., Stojilkovic S.S., Catt K.J. (2000). Local Regulation of Gonadotroph Function by Pituitary Gonadotropin-Releasing Hormone. Endocrinology.

[27] Gault P.M., Maudsley S., Lincoln G.A. (2003). Evidence That Gonadotropin-Releasing Hormone II Is Not a Physiological Regulator of Gonadotropin Secretion in Mammals. J. Neuroendocrinol..

[28] Hassanein E.M., Szelényi Z., Szenci O. (2024). Gonadotropin-Releasing Hormone (GnRH) and Its Agonists in Bovine Reproduction I: Structure, Biosynthesis, Physiological Effects, and Its Role in Estrous Synchronization. Animals.

[29] Rizvi S.F.A., Zhang H., Fang Q. (2024). Engineering Peptide Drug Therapeutics through Chemical Conjugation and Implication in Clinics. Med. Res. Rev..

[30] Maggi R. (2016). Physiology of Gonadotropin-Releasing Hormone (Gnrh): Beyond the Control of Reproductive Functions. MOJ Anat. Physiol..

[31] He Y., Zhang L., Song C. (2011). PEGylated Liposomes Modified with LHRH Analogs for Tumor Targeting. J. Control. Release.

[32] Schally A. (1999). V Luteinizing Hormone-Releasing Hormone Analogs: Their Impact on the Control of Tumorigenesis. Peptides.

[33] Li X., Shen B., Chen Q., Zhang X., Ye Y., Wang F., Zhang X. (2016). Antitumor Effects of Cecropin B-LHRH’ on Drug-Resistant Ovarian and Endometrial Cancer Cells. BMC Cancer.

[34] Chatzaki E., Bax C.M., Eidne K.A., Anderson L., Grudzinskas J.G., Gallagher C.J. (1996). The Expression of Gonadotropin-Releasing Hormone and Its Receptor in Endometrial Cancer, and Its Relevance as an Autocrine Growth Factor. Cancer Res..

[35] Shore N.D., Abrahamsson P.-A., Anderson J., Crawford E.D., Lange P. (2013). New Considerations for ADT in Advanced Prostate Cancer and the Emerging Role of GnRH Antagonists. Prostate Cancer Prostatic Dis..

[36] Cook T., Sheridan W.P. (2000). Development of GnRH Antagonists for Prostate Cancer: New Approaches to Treatment. Oncologist.

[37] Kang S.K., Choi K.-C., Cheng K.W., Nathwani P.S., Auersperg N., Leung P.C.K. (2000). Role of Gonadotropin-Releasing Hormone as an Autocrine Growth Factor in Human Ovarian Surface Epithelium1. Endocrinology.

[38] Völker P., Gründker C., Schmidt O., Schulz K.D., Emons G. (2002). Expression of receptors for luteinizing hormone-releasing hormone in human ovarian and endometrial cancers: Frequency, autoregulation, and correlation with direct antiproliferative activity of luteinizing hormone-releasing hormone analogues. Am. J. Obstet. Gynecol..

[39] Gründker C., Emons G. (2021). Role of Gonadotropin-Releasing Hormone (GnRH) in Ovarian Cancer. Cells.

[40] Clarke I.J. (2011). Control of GnRH Secretion: One Step Back. Front. Neuroendocrinol..

[41] Limonta P., Marelli M.M., Moretti R.M. (2001). LHRH Analogues as Anticancer Agents: Pituitary and Extrapituitary Sites of Action. Expert Opin. Investig. Drugs.

[42] Nagy A., Schally A.V. (2005). Targeting of Cytotoxic Luteinizing Hormone-Releasing Hormone Analogs to Breast, Ovarian, Endometrial, and Prostate Cancers. Biol. Reprod..

[43] Cheng C.K., Leung P.C.K. (2005). Molecular Biology of Gonadotropin-Releasing Hormone (GnRH)-I, GnRH-II, and Their Receptors in Humans. Endocr. Rev..

[44] Jaszberenyi M., Schally A.V., Block N.L., Nadji M., Vidaurre I., Szalontay L., Rick F.G. (2013). Inhibition of U-87 MG Glioblastoma by AN-152 (AEZS-108), a Targeted Cytotoxic Analog of Luteinizing Hormone-Releasing Hormone. Oncotarget.

[45] Sion-Vardi Ν., Kaneti J., Segal-Abramson T., Giat J., Levy J., Sharoni Y. (1992). Gonadotropin-Releasing Hormone Specific Binding Sites in Normal and Malignant Renal Tissue. J. Urol..

[46] Pati D., Habibi H.R. (1995). Inhibition of Human Hepatocarcinoma Cell Proliferation by Mammalian and Fish Gonadotropin-Releasing Hormones. Endocrinology.

[47] Moretti R.M., Montagnani Marelli M., Van Groeninghen J.C., Limonta P. (2002). Locally Expressed LHRH Receptors Mediate the Oncostatic and Antimetastatic Activity of LHRH Agonists on Melanoma Cells. J. Clin. Endocrinol. Metab..

[48] Friess H., Büchler M., Kiesel L., Krüger M., Beger H.G. (1991). LH-RH Receptors in the Human Pancreas. Basis for Antihormonal Treatment in Ductal Carcinoma of the Pancreas. Int. J. Pancreatol..

[49] Roy J., Kaake M., Low P.S. (2019). Small Molecule Targeted NIR Dye Conjugate for Imaging LHRH Receptor Positive Cancers. Oncotarget.

[50] Ahmad Z., Shepherd J.H., Shepherd D.V., Ghose S., Kew S.J., Cameron R.E., Best S.M., Brooks R.A., Wardale J., Rushton N. (2015). Effect of 1-Ethyl-3-(3-Dimethylaminopropyl) Carbodiimide and N-Hydroxysuccinimide Concentrations on the Mechanical and Biological Characteristics of Cross-Linked Collagen Fibres for Tendon Repair. Regen. Biomater..

[51] He R., Finan B., Mayer J.P., DiMarchi R.D. (2019). Peptide Conjugates with Small Molecules Designed to Enhance Efficacy and Safety. Molecules.

[52] Bentivoglio V., Nayak P., Varani M., Lauri C., Signore A. (2023). Methods for Radiolabeling Nanoparticles (Part 3): Therapeutic Use. Biomolecules.

[53] Bentivoglio V., Varani M., Lauri C., Ranieri D., Signore A. (2022). Methods for Radiolabelling Nanoparticles: PET Use (Part 2). Biomolecules.

[54] Varani M., Bentivoglio V., Lauri C., Ranieri D., Signore A. (2022). Methods for Radiolabelling Nanoparticles: SPECT Use (Part 1). Biomolecules.

[55] Jadhav K., Abhang A., Kole E.B., Gadade D., Dusane A., Iyer A., Sharma A., Rout S.K., Gholap A.D., Naik J. (2025). Peptide–Drug Conjugates as Next-Generation Therapeutics: Exploring the Potential and Clinical Progress. Bioengineering.

[56] Bajusz S., Janaky T., Csernus V.J., Bokser L., Fekete M., Srkalovic G., Redding T.W., Schally A. (1989). V Highly Potent Metallopeptide Analogues of Luteinizing Hormone-Releasing Hormone. Proc. Natl. Acad. Sci. USA.

[57] Nagy A., Schally A.V., Armatis P., Szepeshazi K., Halmos G., Kovacs M., Zarandi M., Groot K., Miyazaki M., Jungwirth A. (1996). Cytotoxic Analogs of Luteinizing Hormone-Releasing Hormone Containing Doxorubicin or 2-Pyrrolinodoxorubicin, a Derivative 500-1000 Times More Potent. Proc. Natl. Acad. Sci. USA.

[58] Westphalen S., Kotulla G., Kaiser F., Krauss W., Werning G., Elsasser H.P., Nagy A., Schulz K.D., Grundker C., Schally A.V. (2000). Receptor Mediated Antiproliferative Effects of the Cytotoxic LHRH Agonist AN-152 in Human Ovarian and Endometrial Cancer Cell Lines. Int. J. Oncol..

[59] Gründker C., Völker P., Griesinger F., Ramaswamy A., Nagy A., Schally A.V., Emons G. (2002). Antitumor Effects of the Cytotoxic Luteinizing Hormone–Releasing Hormone Analog AN-152 on Human Endometrial and Ovarian Cancers Xenografted into Nude Mice. Am. J. Obstet. Gynecol..

[60] Günthert A.R., Gründker C., Bongertz T., Schlott T., Nagy A., Schally A.V., Emons G. (2004). Internalization of Cytotoxic Analog AN-152 of Luteinizing Hormone-Releasing Hormone Induces Apoptosis in Human Endometrial and Ovarian Cancer Cell Lines Independent of Multidrug Resistance-1 (MDR-1) System. Am. J. Obstet. Gynecol..

[61] Arencibia J., Schally A., Krupa M., Bajo A., Nagy A., Szepeshazi K., Plonowski A. (2001). Targeting of Doxorubicin to ES-2 Human Ovarian Cancers in Nude Mice by Linking to an Analog of Luteinizing Hormone-Releasing Hormone Improves Its Effectiveness. Int. J. Oncol..

[62] Reutter M., Emons G., Gründker C. (2013). Starving Tumors: Inhibition of Glycolysis Reduces Viability of Human Endometrial and Ovarian Cancer Cells and Enhances Antitumor Efficacy of GnRH Receptor-Targeted Therapies. Int. J. Gynecol. Cancer.

[63] Arencibia J.M., Schally A.V., Halmos G., Nagy A., Kiaris H. (2001). In Vitro Targeting of a Cytotoxic Analog of Luteinizing Hormone-Releasing Hormone AN-207 to ES-2 Human Ovarian Cancer Cells as Demonstrated by Microsatellite Analyses. Anticancer Drugs.

[64] Arencibia J.M., Bajo A.M., Schally A.V., Krupa M., Chatzistamou I., Nagy A. (2002). Effective Treatment of Experimental ES-2 Human Ovarian Cancers with a Cytotoxic Analog of Luteinizing Hormone-Releasing Hormone AN-207. Anticancer Drugs.

[65] Buchholz S., Keller G., Schally A.V., Halmos G., Hohla F., Heinrich E., Koester F., Baker B., Engel J.B. (2006). Therapy of Ovarian Cancers with Targeted Cytotoxic Analogs of Bombesin, Somatostatin, and Luteinizing Hormone-Releasing Hormone and Their Combinations. Proc. Natl. Acad. Sci. USA.

[66] Ma S., Pradeep S., Villar-Prados A., Wen Y., Bayraktar E., Mangala L.S., Kim M.S., Wu S.Y., Hu W., Rodriguez-Aguayo C. (2019). GnRH-R–Targeted Lytic Peptide Sensitizes *BRCA* Wild-Type Ovarian Cancer to PARP Inhibition. Mol. Cancer Ther..

[67] Dharap S. (2003). Molecular Targeting of Drug Delivery Systems to Ovarian Cancer by BH3 and LHRH Peptides. J. Control. Release.

[68] Chandna P., Khandare J.J., Ber E., Rodriguez-Rodriguez L., Minko T. (2010). Multifunctional Tumor-Targeted Polymer-Peptide-Drug Delivery System for Treatment of Primary and Metastatic Cancers. Pharm. Res..

[69] Khandare J.J., Chandna P., Wang Y., Pozharov V.P., Minko T. (2006). Novel Polymeric Prodrug with Multivalent Components for Cancer Therapy. J. Pharmacol. Exp. Ther..

[70] Yao H., Xu Z., Li C., Tse M.-K., Tong Z., Zhu G. (2019). Synthesis and Cytotoxic Study of a Platinum(IV) Anticancer Prodrug with Selectivity toward Luteinizing Hormone-Releasing Hormone (LHRH) Receptor-Positive Cancer Cells. Inorg. Chem..

[71] Markatos C., Biniari G., Chepurny O.G., Karageorgos V., Tsakalakis N., Komontachakis G., Vlata Z., Venihaki M., Holz G.G., Tselios T. (2024). Cytotoxic Activity of Novel GnRH Analogs Conjugated with Mitoxantrone in Ovarian Cancer Cells. Molecules.

[72] Verschraegen C.F., Westphalen S., Hu W., Loyer E., Kudelka A., Völker P., Kavanagh J., Steger M., Schulz K.-D., Emons G. (2003). Phase II Study of Cetrorelix, a Luteinizing Hormone-Releasing Hormone Antagonist in Patients with Platinum-Resistant Ovarian Cancer. Gynecol. Oncol..

[73] Emons G., Gorchev G., Sehouli J., Wimberger P., Stähle A., Hanker L., Hilpert F., Sindermann H., Gründker C., Harter P. (2014). Efficacy and Safety of AEZS-108 (INN: Zoptarelin Doxorubicin Acetate) an LHRH Agonist Linked to Doxorubicin in Women with Platinum Refractory or Resistant Ovarian Cancer Expressing LHRH Receptors: A Multicenter Phase II Trial of the Ago-Study Group (AGO GYN 5). Gynecol. Oncol..

[74] Curtis K.K., Sarantopoulos J., Northfelt D.W., Weiss G.J., Barnhart K.M., Whisnant J.K., Leuschner C., Alila H., Borad M.J., Ramanathan R.K. (2014). Novel LHRH-Receptor-Targeted Cytolytic Peptide, EP-100: First-in-Human Phase I Study in Patients with Advanced LHRH-Receptor-Expressing Solid Tumors. Cancer Chemother. Pharmacol..

[75] Chelariu-Raicu A., Nick A., Urban R., Gordinier M., Leuschner C., Bavisotto L., Molin G.Z.D., Whisnant J.K., Coleman R.L. (2021). A Multicenter Open-Label Randomized Phase II Trial of Paclitaxel plus EP-100, a Novel LHRH Receptor-Targeted, Membrane-Disrupting Peptide, versus Paclitaxel Alone for Refractory or Recurrent Ovarian Cancer. Gynecol. Oncol..

[76] Miller D.S., Scambia G., Bondarenko I., Westermann A.M., Oaknin A., Oza A.M., Lisyanskaya A.S., Vergote I., Wenham R.M., Temkin S.M. (2018). ZoptEC: Phase III randomized controlled study comparing zoptarelin with doxorubicin as second-line therapy for locally advanced, recurrent, or metastatic endometrial cancer (NCT01767155). Int. J. Gynecol. Cancer.

[77] C L. (2017). Targeted Oncolytic Peptide for Treatment of Ovarian Cancers. Int. J. Cancer Res. Mol. Mech..

[78] Wu M., Huang W., Yang N., Liu Y. (2022). Learn from Antibody–Drug Conjugates: Consideration in the Future Construction of Peptide-Drug Conjugates for Cancer Therapy. Exp. Hematol. Oncol..

[79] Hoppenz P., Els-Heindl S., Beck-Sickinger A.G. (2020). Peptide-Drug Conjugates and Their Targets in Advanced Cancer Therapies. Front. Chem..

[80] Maggi R., Cariboni A.M., Marelli M.M., Moretti R.M., Andre V., Marzagalli M., Limonta P. (2016). GnRH and GnRH receptors in the pathophysiology of the human female reproductive system. Hum. Reprod. Update.

[81] Vankadara S., Ke Z., Wang S., Foo S.Y., Gunaratne J., Lee M.A., Koh X., Chia C.S.B. (2024). Cytotoxic Activity and Cell Specificity of a Novel LHRH Peptide Drug Conjugate, *D*-Cys6-LHRH Vedotin, against Ovarian Cancer Cell Lines. Chem. Biol. Drug Des..

[82] Zhang J., Ding H., Zhang F., Xu Y., Liang W., Huang L. (2023). New Trends in Diagnosing and Treating Ovarian Cancer Using Nanotechnology. Front. Bioeng. Biotechnol..

[83] Pan Q., Tian J., Zhu H., Hong L., Mao Z., Oliveira J.M., Reis R.L., Li X. (2020). Tumor-Targeting Polycaprolactone Nanoparticles with Codelivery of Paclitaxel and IR780 for Combinational Therapy of Drug-Resistant Ovarian Cancer. ACS Biomater. Sci. Eng..

[84] Qin Y., Song Q.-G., Zhang Z.-R., Liu J., Fu Y., He Q., Liu J. (2008). Ovarian Tumor Targeting of Docetaxel-Loaded Liposomes Mediated by Luteinizing Hormone-Releasing Hormone Analogues. Arzneimittelforschung.

[85] Yuan W.-M., Song Q.-G., Zhang Z.-R., Fu Y., Liu J., He Q. (2008). LHRHa Aided Liposomes Targeting to Human Ovarian Tumor Cells: Preparation and Cellular Uptake. Pharmazie.

[86] He Y., Zhang L., Song C. (2010). Luteinizing Hormone-Releasing Hormone Receptor-Mediated Delivery of Mitoxantrone Using LHRH Analogs Modified with PEGylated Liposomes. Int. J. Nanomed..

[87] Ye H., Liu X., Sun J., Zhu S., Zhu Y., Chang S. (2016). Enhanced Therapeutic Efficacy of LHRHa-Targeted Brucea Javanica Oil Liposomes for Ovarian Cancer. BMC Cancer.

[88] Nukolova N.V., Oberoi H.S., Zhao Y., Chekhonin V.P., Kabanov A.V., Bronich T.K. (2013). LHRH-Targeted Nanogels as a Delivery System for Cisplatin to Ovarian Cancer. Mol. Pharm..

[89] Zhu X., Yan S., Xiao F., Xue M. (2021). PLGA Nanoparticles Delivering CPT-11 Combined with Focused Ultrasound Inhibit Platinum Resistant Ovarian Cancer. Transl. Cancer Res..

[90] Taheri A., Dinarvand R., Atyabi F., Ahadi F., Nouri F.S., Ghahremani M.H., Ostad S.N., Borougeni A.T., Mansoori P. (2011). Enhanced Anti-Tumoral Activity of Methotrexate-Human Serum Albumin Conjugated Nanoparticles by Targeting with Luteinizing Hormone-Releasing Hormone (LHRH) Peptide. Int. J. Mol. Sci..

[91] Qi N., Zhou X., Ma N., Zhang J., Wang Z., Zhang X., Li A. (2024). Integrin Avβ3 and LHRH Receptor Double Directed Nano-Analogue Effective Against Ovarian Cancer in Mice Model. Int. J. Nanomed..

[92] Wang R., Hu X., Yue J., Zhang W., Cai L., Xie Z., Huang Y., Jing X. (2013). Luteinizing-Hormone-Releasing-Hormone-Containing Biodegradable Polymer Micelles for Enhanced Intracellular Drug Delivery. J. Mater. Chem. B.

[93] Pu C., Chang S., Sun J., Zhu S., Liu H., Zhu Y., Wang Z., Xu R.X. (2014). Ultrasound-Mediated Destruction of LHRHa-Targeted and Paclitaxel-Loaded Lipid Microbubbles for the Treatment of Intraperitoneal Ovarian Cancer Xenografts. Mol. Pharm..

[94] Xu C., Yuan Z., Kohler N., Kim J., Chung M.A., Sun S. (2009). FePt Nanoparticles as an Fe Reservoir for Controlled Fe Release and Tumor Inhibition. J. Am. Chem. Soc..

[95] Gao Y., Chen S., Li W., Wang H., Xiao K., Wu L., Li Y., Li H., Li H., Zhu Y. (2018). An Experimental Study of Ovarian Cancer Imaging and Therapy by Paclitaxel-Loaded Phase-Transformation Lipid Nanoparticles Combined with Low-Intensity Focused Ultrasound. Biochem. Biophys. Res. Commun..

[96] Singh P., Duraisamy K., Raitmayr C., Sharma K.S., Korzun T., Singh K., Moses A.S., Yamada K., Grigoriev V., Demessie A.A. (2025). Precision-Engineered Cobalt-Doped Iron Oxide Nanoparticles: From Octahedron Seeds to Cubical Bipyramids for Enhanced Magnetic Hyperthermia. Adv. Funct. Mater..

[97] Kim S.H., Jeong J.H., Lee S.H., Kim S.W., Park T.G. (2008). LHRH Receptor-Mediated Delivery of SiRNA Using Polyelectrolyte Complex Micelles Self-Assembled from SiRNA-PEG-LHRH Conjugate and PEI. Bioconjug Chem..

[98] Shah V., Taratula O., Garbuzenko O.B., Taratula O.R., Rodriguez-Rodriguez L., Minko T. (2013). Targeted Nanomedicine for Suppression of CD44 and Simultaneous Cell Death Induction in Ovarian Cancer: An Optimal Delivery of SiRNA and Anticancer Drug. Clin. Cancer Res..

[99] Schumann C., Taratula O., Khalimonchuk O., Palmer A.L., Cronk L.M., Jones C.V., Escalante C.A., Taratula O. (2015). ROS-Induced Nanotherapeutic Approach for Ovarian Cancer Treatment Based on the Combinatorial Effect of Photodynamic Therapy and DJ-1 Gene Suppression. Nanomedicine.

[100] Schumann C., Chan S., Khalimonchuk O., Khal S., Moskal V., Shah V., Alani A.W.G., Taratula O., Taratula O. (2016). Mechanistic Nanotherapeutic Approach Based on SiRNA-Mediated DJ-1 Protein Suppression for Platinum-Resistant Ovarian Cancer. Mol. Pharm..

[101] Schumann C., Chan S., Millar J.A., Bortnyak Y., Carey K., Fedchyk A., Wong L., Korzun T., Moses A.S., Lorenz A. (2018). Intraperitoneal Nanotherapy for Metastatic Ovarian Cancer Based on SiRNA-Mediated Suppression of DJ-1 Protein Combined with a Low Dose of Cisplatin. Nanomedicine.

[102] Patil M.L., Zhang M., Taratula O., Garbuzenko O.B., He H., Minko T. (2009). Internally Cationic Polyamidoamine PAMAM-OH Dendrimers for SiRNA Delivery: Effect of the Degree of Quaternization and Cancer Targeting. Biomacromolecules.

[103] Yu C., Ding B., Zhang X., Deng X., Deng K., Cheng Z., Xing B., Jin D., Ma P., Lin J. (2018). Targeted Iron Nanoparticles with Platinum-(IV) Prodrugs and Anti-EZH2 SiRNA Show Great Synergy in Combating Drug Resistance in Vitro and in Vivo. Biomaterials.

[104] Chang S., Guo J., Sun J., Zhu S., Yan Y., Zhu Y., Li M., Wang Z., Xu R.X. (2013). Targeted Microbubbles for Ultrasound Mediated Gene Transfection and Apoptosis Induction in Ovarian Cancer Cells. Ultrason. Sonochem.

[105] Zhao M., Liu Y., Hsieh R.S., Wang N., Tai W., Joo K.-I., Wang P., Gu Z., Tang Y. (2014). Clickable Protein Nanocapsules for Targeted Delivery of Recombinant P53 Protein. J. Am. Chem. Soc..

[106] Alatise K.L., Samec T., Coffin C., Gilmore S., Hazelton A., Xia R., Jones C., Alexander-Bryant A. (2025). Multifunctional Tandem Peptide Mediates Targeted SiRNA Delivery to Ovarian Cancer Cells. ACS Appl. Bio Mater..

[107] Garbuzenko O.B., Sapiezynski J., Girda E., Rodriguez-Rodriguez L., Minko T. (2024). Personalized Versus Precision Nanomedicine for Treatment of Ovarian Cancer. Small.

[108] Wang Y., Wang Y., Chen G., Li Y., Xu W., Gong S. (2017). Quantum-Dot-Based Theranostic Micelles Conjugated with an Anti-EGFR Nanobody for Triple-Negative Breast Cancer Therapy. ACS Appl. Mater. Interfaces.

[109] Biswas Majee S., Avlani D., Kumar A., Bera R. (2025). Harnessing Nanotheranostics for the Management of Breast and Ovarian Cancer. Acad. Nano Sci. Mater. Technol..

[110] Vishwasrao H.M., Master A.M., Seo Y.G., Liu X.M., Pothayee N., Zhou Z., Yuan D., Boska M.D., Bronich T.K., Davis R.M. (2016). Luteinizing Hormone Releasing Hormone-Targeted Cisplatin-Loaded Magnetite Nanoclusters for Simultaneous MR Imaging and Chemotherapy of Ovarian Cancer. Chem. Mater..

[111] Taratula O., Schumann C., Naleway M.A., Pang A.J., Chon K.J., Taratula O. (2013). A Multifunctional Theranostic Platform Based on Phthalocyanine-Loaded Dendrimer for Image-Guided Drug Delivery and Photodynamic Therapy. Mol. Pharm..

[112] Taratula O., Taratula O., Patel M., Schumann C., Naleway M., He H., Pang A. (2015). Phthalocyanine-Loaded Graphene Nanoplatform for Imaging-Guided Combinatorial Phototherapy. Int. J. Nanomed..

[113] Lin C.-J., Kuan C.-H., Wang L.-W., Wu H.-C., Chen Y., Chang C.-W., Huang R.-Y., Wang T.-W. (2016). Integrated Self-Assembling Drug Delivery System Possessing Dual Responsive and Active Targeting for Orthotopic Ovarian Cancer Theranostics. Biomaterials.

[114] Savla R., Garbuzenko O.B., Chen S., Rodriguez-Rodriguez L., Minko T. (2014). Tumor-Targeted Responsive Nanoparticle-Based Systems for Magnetic Resonance Imaging and Therapy. Pharm. Res..

[115] Kumar D., Moghiseh M., Chitcholtan K., Mutreja I., Lowe C., Kaushik A., Butler A., Sykes P., Anderson N., Raja A. (2023). LHRH Conjugated Gold Nanoparticles Assisted Efficient Ovarian Cancer Targeting Evaluated via Spectral Photon-Counting CT Imaging: A Proof-of-Concept Research. J. Mater. Chem. B.

[116] Liu A., Li L., Wang Z., Li X., Liang H., Yang J., Nešić M.D., Yang X., Lin Q. (2025). Ultrasmall Au-GRHa Nanosystem for FL/CT Dual-Mode Imaging-Guided Targeting Photothermal Therapy of Ovarian Cancer. Anal. Chem..

[117] Demessie A.A., Park Y., Singh P., Moses A.S., Korzun T., Sabei F.Y., Albarqi H.A., Campos L., Wyatt C.R., Farsad K. (2022). An Advanced Thermal Decomposition Method to Produce Magnetic Nanoparticles with Ultrahigh Heating Efficiency for Systemic Magnetic Hyperthermia. Small Methods.

[118] Schottelius M., Berger S., Poethko T., Schwaiger M., Wester H.-J. (2008). Development of Novel ^68^Ga- and ^18^F-Labeled GnRH-I Analogues with High GnRHR-Targeting Efficiency. Bioconjug. Chem..

